# Effect of Virtual Reality–Based Therapies on Lower Limb Functional Recovery in Stroke Survivors: Systematic Review and Meta-Analysis

**DOI:** 10.2196/72364

**Published:** 2025-07-30

**Authors:** Wenxin Lu, Minglei Shi, Lu Liu, Shu Wang, Wuquan Deng, Yu Ma, Yanzhong Wang

**Affiliations:** 1 Department of Population Health Sciences, School of Life Course and Population Sciences, King's College London, Addison House, Guy's Campus, Newcommen Street, London, SE1 1UL, United Kingdom, 44 7842979595; 2 Department of Intensive Care Medicine, Chongqing Emergency Medical Center, Chongqing University Central Hospital, Chongqing University, Chongqing, China; 3 Department of Endocrinology, School of Medicine, Chongqing Emergency Medical Center, Chongqing University Central Hospital, Chongqing University, Chongqing, China

**Keywords:** virtual reality, stroke rehabilitation, functional recovery, lower extremity, meta-analysis

## Abstract

**Background:**

Virtual reality (VR) therapy has gained attention as a promising intervention in stroke rehabilitation, particularly for its potential to enhance motor function and promote neuroplasticity. However, its specific effects on balance, mobility, and gait remain underexplored.

**Objective:**

This review aims to provide a comprehensive evaluation of the effectiveness of VR therapy on the recovery of lower limb function in stroke survivors.

**Methods:**

Randomized controlled trials comparing VR therapy with conventional therapy were eligible for inclusion. All studies were identified through databases, such as MEDLINE, Embase, PubMed, Cochrane Library, Web of Science, and PsycINFO (up to July 2024). The primary outcomes included balance, assessed using the Berg Balance Scale (BBS), and mobility, measured with the Timed Up and Go Test (TUG) and 10-Meter Walk Test (10-MWT). Secondary outcomes included gait parameters (stride length and step length), the Functional Reach Test (FRT), the Dynamic Gait Index (DGI), and the Falls Efficacy Scale-International (FES-I). RevMan version 5.4 (The Cochrane Collaboration) software was used for the meta-analysis.

**Results:**

A total of 2145 studies were screened, and 24 randomized controlled trials that met the inclusion criteria were included, involving 768 participants. Meta-analysis results showed that VR therapy, compared to conventional therapy, significantly improved BBS (mean difference [MD] 3.29, 95% CI 0.52-6.06; *P*=.02), TUG (MD −1.67, 95% CI −2.89 to −0.46; *P*=.007), and step length (MD 3.59, 95% CI 0.50-6.69; *P*=.02). However, no significant difference was observed between the 2 groups in 10-MWT (MD −0.91, 95% CI −3.33 to 1.50; *P*=.46), stride length (MD 5.63, 95% CI −0.73 to 11.99; *P*=.08), FRT (MD 2.68, 95% CI −0.30 to 5.67; *P*=.08), DGI (MD 1.08, 95% CI −0.41 to 2.58; *P*=.16), and FES-I (MD 0.16, 95% CI −2.92 to 3.24; *P*=.92). In the subgroup analyses, significant improvements in balance and mobility were observed in patients receiving greater than or equal to 20 sessions, with BBS improved by 5.14 points (95% CI 0.43-9.85; *P*=.03) and TUG reduced by 1.98 seconds (95% CI −3.33 to −0.63; *P*=.004). In addition, patients who received VR therapy more than 6 months after stroke showed greater improvements in BBS (MD 1.64, 95% CI 0.14-3.14; *P*=.03), compared to those who received VR therapy between 7 days and 6 months after stroke.

**Conclusions:**

Long-term VR-based therapies are more effective in improving functional ability after stroke. VR therapy has demonstrated significant potential for enhancing lower limb recovery, especially when applied with frequencies of ≥20 sessions.

## Introduction

Stroke is a leading global health issue, causing significant mortality and long-term disability worldwide. Each year, approximately 15 million people experience a stroke, with 5 million left permanently disabled, leading to substantial burdens on individuals, families, and health care systems [1]. Lower limb dysfunction, including impaired balance, gait abnormalities, and muscle weakness, is a common consequence, severely limiting independence and quality of life [2,3]. Prolonged physical impairments often result in secondary complications, such as muscle atrophy and psychological challenges like depression and anxiety, further hindering recovery [4], and may even cause the occurrence of a second stroke [5]. Traditional rehabilitation methods, such as physiotherapy and gait training, aim to restore mobility and function but are often time-intensive, repetitive, low in patient compliance, and reliant on therapist expertise [6,7]. These limitations highlight the need for more engaging and effective approaches to address the diverse rehabilitation needs of stroke survivors [8]. Emerging technologies, particularly virtual reality (VR), offer innovative solutions to enhance stroke rehabilitation by providing immersive and interactive environments for training [9]. According to the level of interaction between the user and the technology, the VR interventions can be broadly classified into three categories [10]: (1) immersive VR (IVR); (2) semi-immersive virtual reality (SIVR); and (3) nonimmersive virtual reality (NIVR). VR-based therapy enables patients to practice activities, namely gait and balance training in safe, realistic VR scenarios, improving engagement and potentially accelerating functional recovery [11]. These advantages have led to the gradual recognition of VR in the field of stroke rehabilitation. Several studies have explored the efficacy of VR-based interventions in improving motor function among stroke survivors, with many findings demonstrating promising outcomes [12-14]. A previous review also highlighted the high potential of VR technology to improve upper limb function through repetitive and task-specific training [15]. The real-time feedback in VR training facilitates immediate movement correction, while its interactivity and immersion establish VR as an effective tool for limb rehabilitation. While its benefits for upper limb rehabilitation are well-documented [16], the evidence supporting VR for lower limb recovery remains limited and requires further exploration. In fact, the restoration of lower limb function can lead to a faster increase in patient autonomy [17], which is significantly important for stroke survivors. Recent studies have shown that VR can help lower limb functional recovery by promoting motor learning and neuroplasticity [18]. For example, a case study of a 58-year-old man [19] showed significant improvements in balance, gait, and lower limb motor function in a patient with right hemiparetic traumatic brain injury after VR treatment. Moreover, VR has been shown to promote cortical reorganization from aberrant ipsilateral to contralateral sensorimotor cortex activation, which is associated with locomotor recovery in patients with chronic stroke [20]. These studies suggested the efficacy of VR interventions in facilitating lower limb functional recovery.

In this study, a systematic review and meta-analysis were performed with the aim of comparing the effectiveness of VR-based interventions to conventional therapies in improving lower limb function among stroke survivors. Although a previous study has addressed a wider range of outcomes [21], including upper- and lower-limb motor function, balance, gait, cognition, and daily function in patients with stroke, this review specifically targets lower-limb functional recovery, providing a more detailed exploration of VR interventions that focus on improving balance, gait, and motor control poststroke. The hypothesis of the study was that VR-based interventions result in superior recovery of lower limb function when compared to traditional therapeutic approaches. This study also wants to provide insights into optimizing rehabilitation approaches to maximize recovery in stroke survivors.

## Methods

### Overview

This review was conducted in accordance with the PRISMA (Preferred Reporting Items for Systematic Reviews and Meta-Analysis) guideline. The systematic review was registered with the International Platform of Registered Systematic Review and Meta-Analysis Protocols (INPLASY; registration number INPLASY2024110101).

### Search Strategy and Selection Criteria

A comprehensive search with no language restrictions was conducted through 6 electronic databases, including MEDLINE, Embase, PubMed, Cochrane Central Register of Controlled Trials (CENTRAL), Web of Science, and PsycINFO. Relevant studies published from database inception to July 2024 were included. Details of the search strategy are provided in Multimedia Appendix 1. Studies were included if they met the following criteria: (1) study design was randomized controlled trials (RCTs), (2) participants were adults (≥18 y) diagnosed with stroke, (3) interventions involved treatment using VR technology, including IVR, SIVR, and NIVR, (4) comparisons were made against routine rehabilitation (conventional therapy), and (5) outcomes assessed lower limb motor function, balance, gait, walking, or mobility. Studies were excluded if they lacked detailed intervention descriptions or outcome indicators, or did not report pre- and postintervention comparisons. In addition, studies combining VR interventions with external neurostimulation techniques were excluded. The detailed criteria are summarized in Table 1.

**Table 1.** Inclusion criteria (based on the Population, Intervention, Control, Outcome, and Study design framework).

Category	Inclusion criteria
Population (P)	Adults (≥18 y) diagnosed with stroke
Intervention (I)	Use of VR^a^ technology, including IVR^b^, SIVR^c^, NIVR^d^
Control (C)	Routine rehabilitation (conventional therapy)
Outcome (O)	Studies assessing lower limb motor function, balance, gait, walking, or mobility
Study design (S)	Randomized controlled trials (RCTs)
Additional criteria	No language restrictions—studies in any language are included Studies must provide detailed intervention descriptions and outcome indicators Studies must report pre- and postintervention comparisons

a VR: virtual reality.

b IVR: immersive virtual reality.

c SIVR: semi-immersive virtual reality.

d NIVR: nonimmersive virtual reality.

### Outcomes

The outcomes of interest were functional recovery of the lower extremities, categorized into primary and secondary outcomes. The primary outcomes included balance, assessed using the Berg Balance Scale (BBS), and mobility, measured by the Timed Up and Go Test (TUG) and the 10-Meter Walk Test (10-MWT). Secondary outcomes included stride and step length, the Functional Reach Test (FRT), the Falls Efficacy Scale (FES), and the Dynamic Gait Index (DGI).

### Data Collection and Extraction

Two reviewers (WL and MS) independently screened the titles and abstracts, removing duplicates and excluding irrelevant studies. The remaining studies were then assessed in full based on predefined inclusion and exclusion criteria. Any disagreements were resolved through discussion, with a third arbiter (YW) involved when necessary. Extracted data included study design, participant characteristics (age, sex, stroke type, and disease duration), intervention details (type, duration, and frequency), and outcome measures (balance, gait speed, and step length). To compare pre- and postintervention data, the mean change (Δmean) was calculated by subtracting the postintervention mean from the preintervention mean. In addition, the change in SD (SD_difference_) was computed using the formula from the Cochrane Handbook [22].

### Bias and Quality Assessment

The Cochrane Risk of Bias (RoB) tool was used to assess the quality of RCTs [23], focusing on sequence generation, allocation concealment, blinding, and outcome reporting. The updated RoB2 tool [24] was used to evaluate five domains: randomization process, deviations from intended interventions, outcome measurement, missing outcome data, and selective reporting. Each domain was rated as low, high, or unclear risk of bias, contributing to an overall judgment for interpreting meta-analysis results.

### Data Synthesis and Statistical Analysis

We performed a meta-analysis using a random-effects model for all continuous variables. Mean differences (MD) and 95% CIs were calculated for primary and secondary outcomes, with *P*<.05 considered statistically significant. *I*² was used to assess heterogeneity. Subgroup analyses were performed based on factors such as time after stroke (≤7 d, >7 d ≤6 mo and >6 mo), VR type (IVR, SIVR, and NIVR) and total frequency of intervention (<20 sessions and ≥20 sessions). We also performed a sensitivity analysis using the latest assessment in each study. RevMan 5.4 (The Cochrane Collaboration) software was used for all analyses and forest plots illustrating the pooled results. Both RevMan 5.4 software and Stata 17.0 (StataCorp) were used for funnel plots and Egger test to assess publication bias.

## Results

### Study Selection

**Figure 1.**
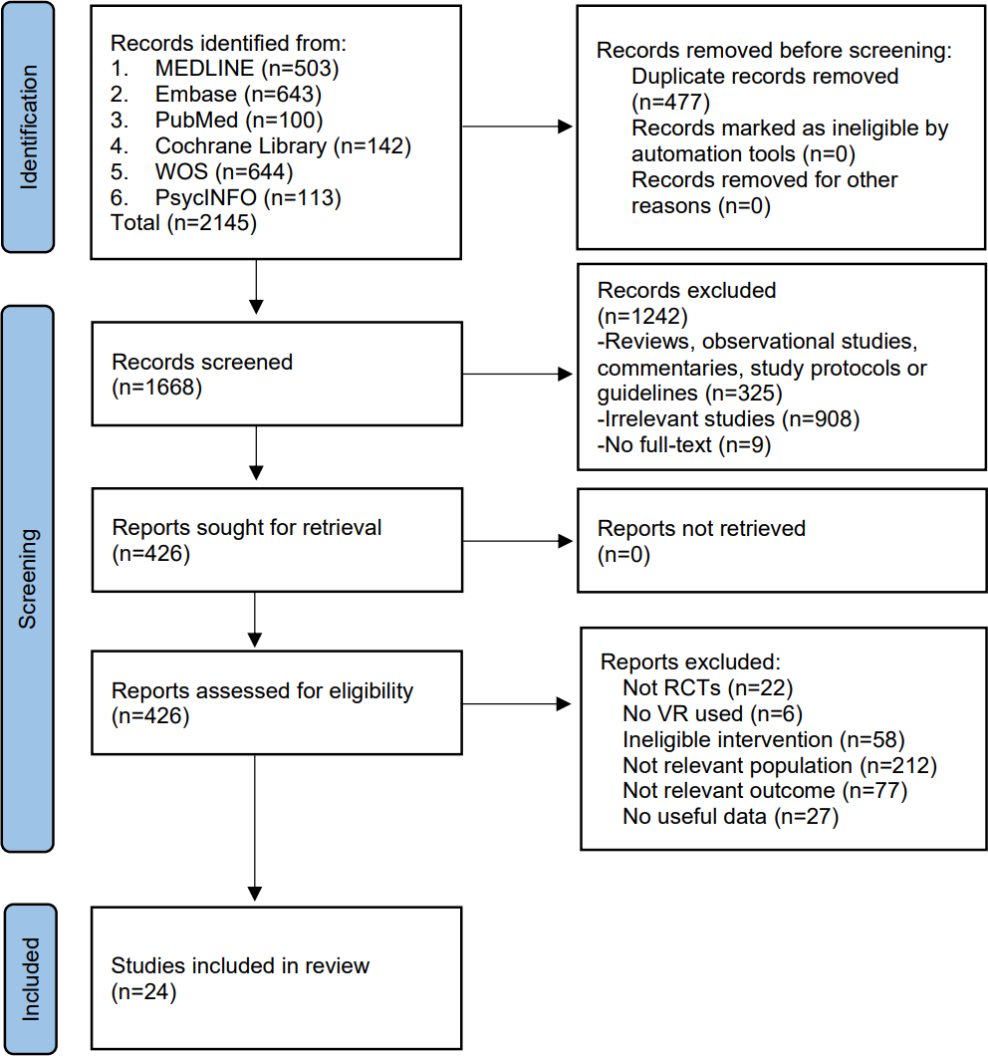
Preferred Reporting Items for Systematic Reviews and Meta-Analysis flowchart of the study selection process. RCT: randomized controlled trial; VR: virtual reality; WOS: Web of Science.

A total of 2145 records were identified through database searches. After removing 477 duplicates, 1668 records were screened based on titles and abstracts, leaving 426 studies for full-text review. Following this review, 24 studies [25-48] were ultimately included in the meta-analysis. The flowchart is presented in Figure 1.

### Characteristics of the Included Studies

A total of 24 RCTs [25-48] comprising 768 participants were included in the analysis. The studies focused on patients with stroke across different recovery stages, with 473 (61.6%) participants targeting individuals more than 6 months poststroke [28] and the remainder focusing on the subacute stage (7 d to 6 mo) [46]. Most studies (16/24, 67%) [25-32,35,37,39-43,48] used a single-blind design, while 3 (13%) studies [36,40,41] used IVR intervention, 7 (29%) studies [29,31,33-35,46,48] used SIVR intervention and 14 (58%) studies [25-28,30,32,37-39,42-45,47] used NIVR intervention. Intervention durations ranged from 3 to 12 weeks, with 10‐40 total sessions and an average of 18‐19 sessions. The most common frequencies were 12 sessions (6 studies [28,29,39,40,43,47]) and 20 sessions (5 studies [31,32,34,35,37]). The baseline characteristics of each study are detailed in Multimedia Appendix 2.

### Outcome of Interest

The meta-analysis results are summarized in Table 2. Three of the outcomes were statistically different.

**Table 2.** Summary of meta-analysis results on the effects of virtual reality–based therapy versus conventional therapy on lower limb function in stroke survivors (randomized controlled trials up to July 2024).

Outcomes	Number of studies	Mean difference (95% CI)	*I*^2a^ (%)
BBS^b^	13	3.29 (0.52 to 6.06)^c^	81
TUG^d^	16	−1.67 (−2.89 to −0.46)^c^	0
10-MWT^e^	7	−0.91 (−3.33 to 1.50)	0
Stride length	5	5.63 (−0.73 to 11.99)	0
Step length	6	3.59 (0.50 to 6.69)^c^	0
FRT^f^	3	2.68 (−0.30 to 5.67)	25
FES-I^g^	2	0.16 (−2.92 to 3.24)	0
DGI^h^	2	1.08 (−0.41 to 2.58)	0

a *I*2: heterogeneity among included studies.

b BBS: Berg Balance Scale.

c Statistically significant.

d TUG: Timed Up and Go Test.

e 10-MWT: 10-Meter Walk Test.

f FRT: Functional Reach Test.

g FES-I: Falls Efficacy Scale-International.

h DGI: Dynamic Gait Index.

### Primary Outcome

#### BBS

**Figure 2.**
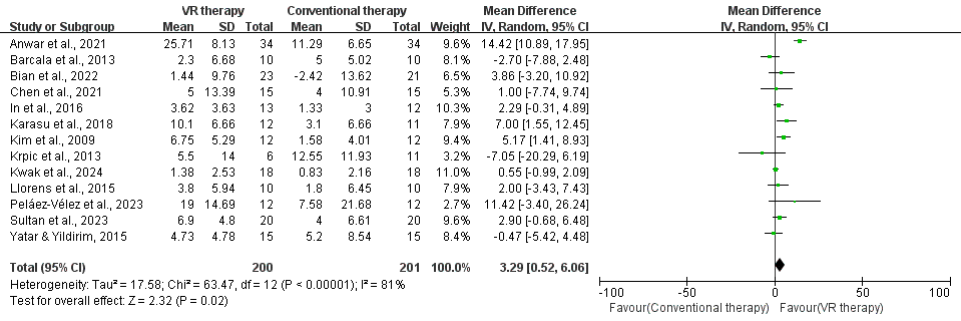
Forest plot for the effect of VR on balance in stroke survivors (measured by the Berg Balance Scale) [25-28,31-34,36,37,41,45,47]. VR: virtual reality.

Meta-analysis of 13 studies [25-28,31-34,36,37,41,45,47] (401 participants) showed VR therapy significantly improved balance in patients with stroke compared to conventional therapy, with a mean BBS score difference of 3.29 (95% CI, 0.52-6.06; *P*=.02). Despite high heterogeneity (*I*²=81%; *P*<.001), the results consistently favored VR therapy (Figure 2).

##### Subgroup Analysis

For patients within 7 days to 6 months after stroke, no significant difference was observed between VR and conventional therapy (MD −0.10, 95% CI −10.18 to 10.18; *P*=.98). In contrast, for patients more than 6 months after stroke, VR therapy demonstrated a significant improvement in BBS compared to conventional treatment (MD 1.64, 95% CI 0.17-3.14; *P*=.03), with low heterogeneity (*I*²=27%). These results suggest that VR therapy may have greater clinical advantages in long-term rehabilitation.

Subgroup analysis based on VR types (IVR, SIVR, and NIVR) revealed varying effects on balance improvement. Although individual subgroups did not achieve statistical significance, the combined effect sizes demonstrated a significant overall benefit, likely due to the increased sample size and cumulative subgroup effects, highlighting the need for cautious interpretation and further research.

**Figure 3.**
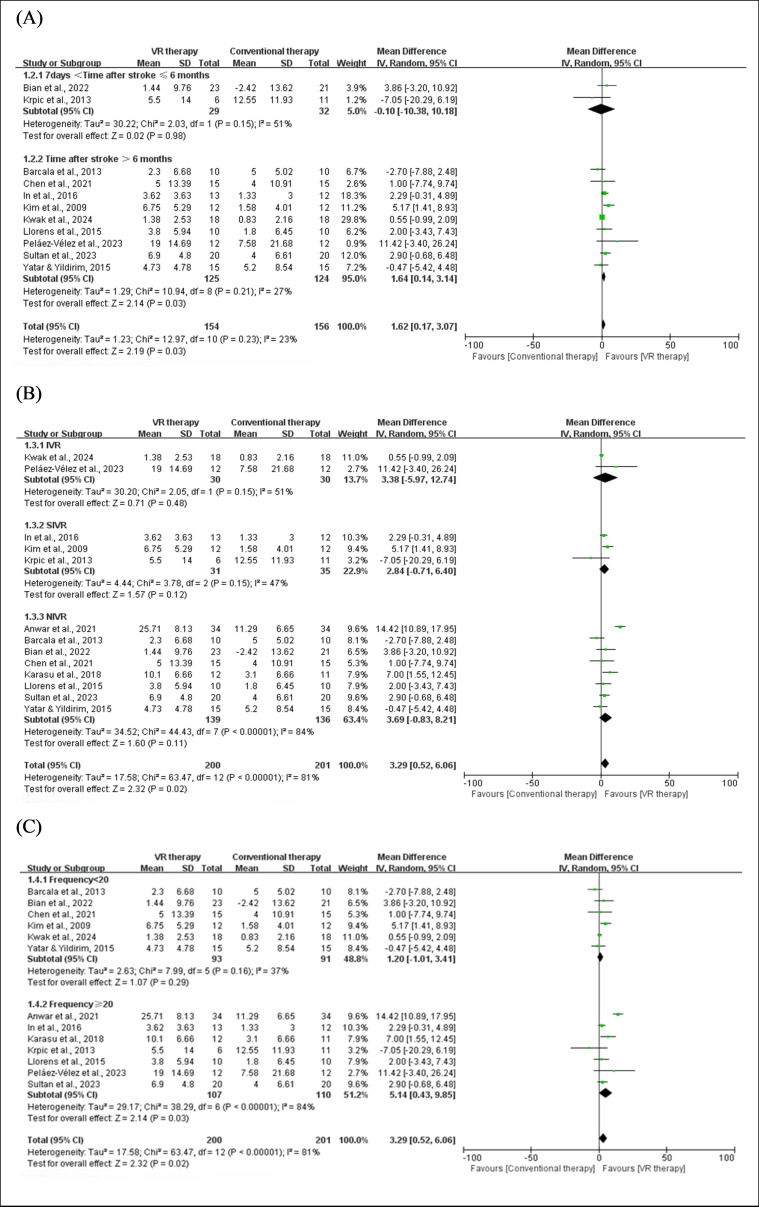
Forest plot for subgroup analysis of VR effects on balance by time after stroke, VR type, and total frequency. A) BBS: time after stroke (1.2.1) 7 days <time after stroke ≤6 months [27,34]; (1.2.2) Time after stroke >6 months [26,28,31,33,36,37,41,45,47]. (B) BBS: VR type (1.3.1) immersive virtual reality (IVR) [36,41]; (1.3.2) semi-immersive virtual reality (SIVR) [31,33,34]; (13.3) nonimmersive virtual reality (NIVR) [25-28,32,37,45,47]. (C) BBS: Total frequency (1.4.1); Frequency <20 [26-28,33,36,47] (1.4.2); Frequency ≥20 [25,31,32,34,37,41,45]. BBS: Berg Balance Scale; VR: virtual reality.

Subgroup analysis based on intervention frequency showed that VR therapy significantly improved balance when the total sessions were ≥20 (MD 5.14, 95% CI 0.43-9.85; *P*=.03). In contrast, studies with <20 sessions showed no significant improvement (MD 1.20, 95% CI −1.01 to 3.41; *P*=.29). These results highlight that a higher intervention frequency (≥20 sessions) is crucial for achieving significant balance improvements in patients with stroke. The above results are demonstrated in Figure 3.

##### Sensitivity Analysis

Five of the included studies [25-27,32,47] used Nintendo Wii devices, 2 studies [41,45] used Xbox Kinect, and the other 6 studies [28,31,33,34,36,37] each used a different device. The pooled MD was 4.54 (95% CI −2.54 to 11.62; *P*=.21) seconds. Heterogeneity was low (*I*^2^=90% *P*<.05) in studies using Nintendo Wii devices. The pooled MD was 4.00 (95% CI −1.60 to 9.61; *P*=.16)seconds. Heterogeneity was low (*I*^2^=17%; *P*=.27) in studies using Xbox Kinect devices. The pooled MD was 1.79 (95% CI −0.00 to 3.59; *P*=.05) seconds. Heterogeneity was low (*I*^2^=30%; *P*=.21) in studies using other devices (Multimedia Appendix 3).

### TUG

**Figure 4.**
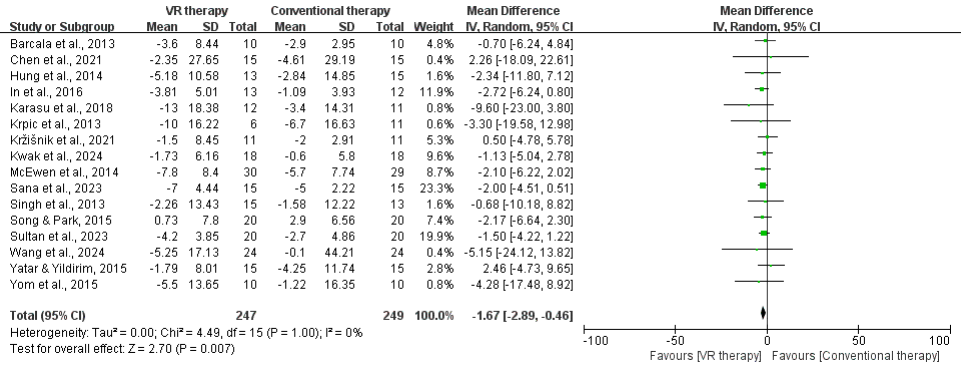
Forest plot for the effect of VR on mobility in stroke survivors (measured by Timed Up and Go Test) [26,28,30-32,34-36,38,42-48]. VR: virtual reality.

Meta-analysis of 16 studies (496 participants) showed VR therapy significantly improved lower limb mobility in patients with stroke compared to conventional therapy, with a mean TUG score difference of −1.67 (95% CI, −2.89 to −0.46; *P*=.007) seconds. Heterogeneity was low (*I*²=0%; *P*=1.00), indicating consistent and reliable results favoring VR therapy (Figure 4).

VR therapy significantly improved TUG scores in patients >6 months poststroke (MD −1.53 s, 95% CI −3.08 to −0.02; *P*=.05). In contrast, no significant improvement was observed in patients 7 days to 6 months poststroke (MD −1.62 s, 95% CI −3.85 to 0.61; *P*=.15). These findings underscore the greater effectiveness of VR therapy in improving mobility in patients with chronic stroke.

NIVR significantly improved TUG scores compared to conventional therapy (MD −1.67 s, 95% CI −3.10 to −0.23; *P*=.02) with no heterogeneity. In contrast, neither IVR (MD −1.13 s, 95% CI −5.04 to 2.78; *P*=.57) nor SIVR (MD −1.96 s, 95% CI −4.75 to 0.82; *P*=.17) showed significant effects, highlighting the superior efficacy of nonimmersive VR in improving functional mobility in patients with stroke.

**Figure 5.**
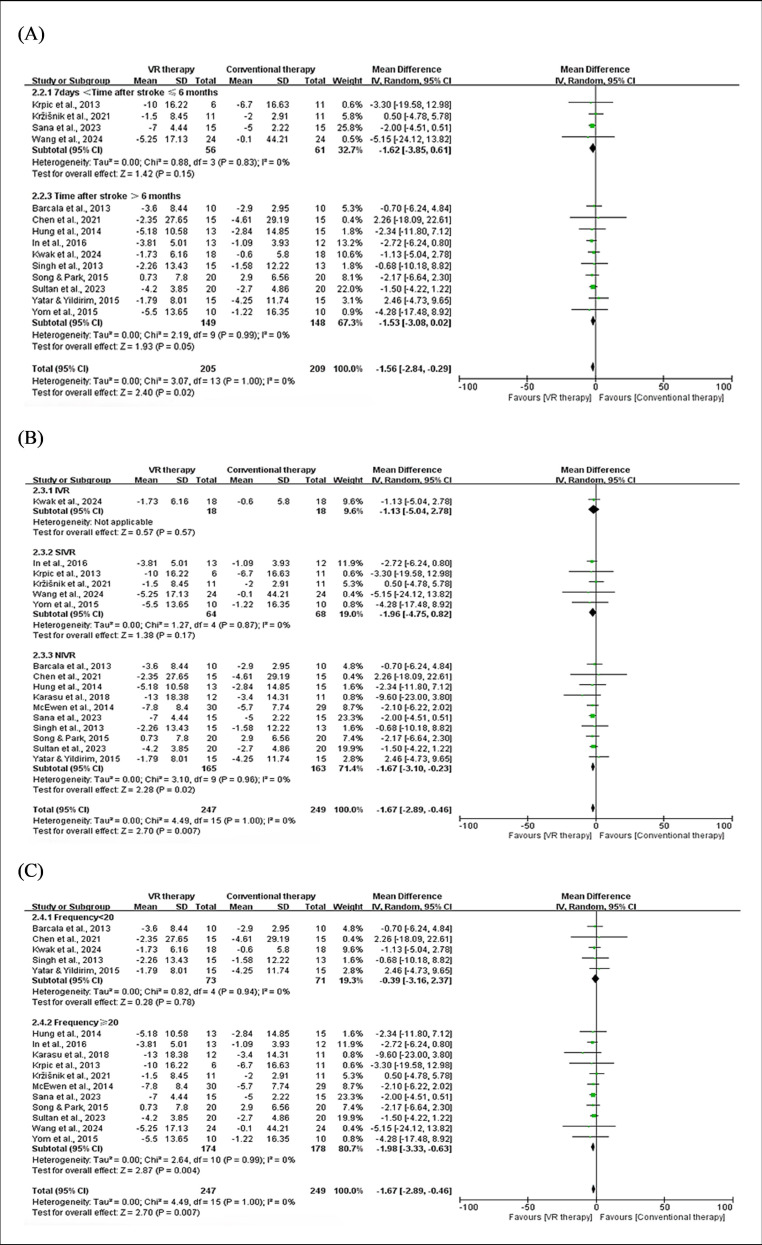
Forest plot for subgroup analysis of VR effects on mobility by time after stroke, VR type, and total frequency. (A) TUG: time after stroke (2.2.1) 7 days <time after stroke ≤6 months [34,35,42,46]; (2.2.3) time after stroke >6 months [26,28,30,31,36,43-45,47,48]. (B) TUG: VR type (2.3.1) immersive virtual reality (IVR) [36]; (2.3.2) semi-immersive virtual reality (SIVR) [31,34,35,46,48], (2.3.3); nonimmersive virtual reality (NIVR) [26,28,30,32,38,42-45,47]. (C) TUG: total frequency (2.4.1) Frequency <20 [26,28,36,43,47]; (2.4.2) Frequency ≥20 [30-32,34,35,38,42,44-46,48]. TUG: Timed Up and Go Test; VR: virtual reality.

A total frequency of ≥20 sessions significantly improved TUG performance compared to conventional therapy (MD −1.98 s, 95% CI −3.33 to −0.63; *P*=.004) with no heterogeneity. In contrast, <20 sessions showed no significant effect (MD –0.39 s, 95% CI −3.16 to 2.37; *P*=.78). Emphasizing the enhanced efficacy of higher-frequency VR therapy in promoting lower limb mobility improvement. The above results are demonstrated in Figure 5.

### 10-MWT

**Figure 6.**
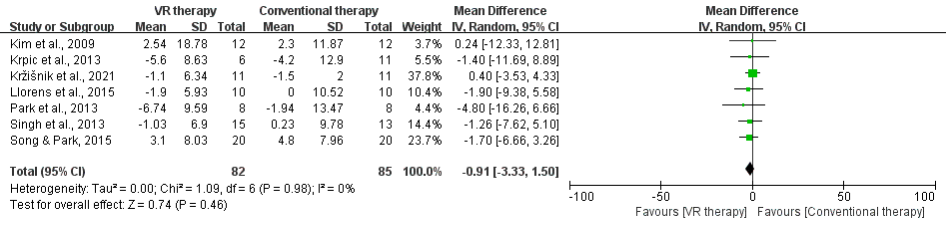
Forest plot for the effect of VR on gait speed in stroke survivors (measured by 10-Meter Walk Test) [33-35,37,40,43,44]. VR: virtual reality; 10-MWT: 10-Meter Walk Test.

Seven studies [33-35,37,40,43,44] (167 participants) showed no significant difference in gait speed improvement between VR therapy and conventional treatment, with a mean 10-MWT difference of −0.91 (95% CI −3.33 to 1.50; *P*=.46) seconds. Heterogeneity was low (*I*^2^=0%; *P*=.98), indicating high consistency across studies, but the overall results did not favor VR therapy (Figure 6). Meanwhile, subgroup analysis revealed that VR therapy did not significantly improve gait speed across different poststroke durations, VR types, or treatment frequencies, indicating limited efficacy in enhancing 10-MWT outcomes. Forest plots for these results are shown in Figures S1-S3 in Multimedia Appendix 4.

### Secondary Outcome

#### Stride Length

Five studies [27,33,36,39,40,48] assessing stride length showed no significant improvement with VR therapy compared to conventional treatment (*P*=.08), despite a positive trend. The forest plot is in Figure S4 in Multimedia Appendix 4.

#### Step Length

**Figure 7.**
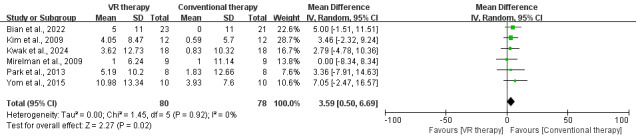
Forest plot for the effect of VR on step length [27,33,36,39,40,48]. VR: virtual reality

Six studies [27,33,36,39,40,48] demonstrated that VR therapy significantly improved step length, with an MD of 3.59 (95% CI 0.50-6.69; *P*=.02). Low heterogeneity (*I*²=0%) indicated high consistency and reliability across studies, highlighting the superior effectiveness of VR therapy in enhancing step length (Figure 7).

#### FRT, DGI, and FES-I

Regarding the FRT and DGI, which assess dynamic balance, no significant differences were observed between VR therapy and conventional treatment. Similarly, for the FES-I, VR therapy did not demonstrate a significant advantage in reducing the fear of falling. However, the limited number of studies may have reduced the ability to detect a true effect, highlighting the need for further research to explore the potential benefits of VR therapy in improving dynamic balance and gait safety. Forest plots for these results are presented in Figures S5-S7 in Multimedia Appendix 4.

### Risk of Bias Assessment and Publication Bias

**Figure 8.**
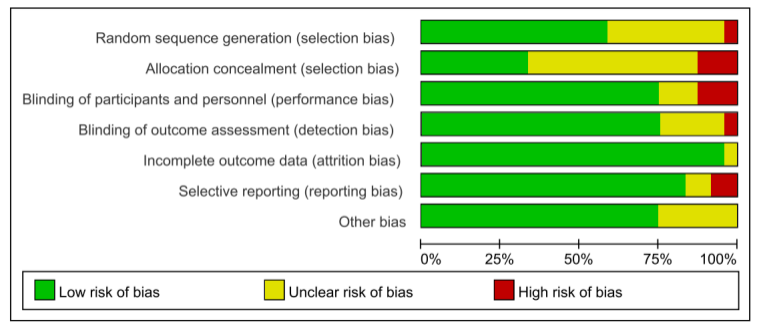
Risk of bias assessment for included studies.

More than 50% (14/24) of the studies [25-27,29-32,35,37,38,41,42,44,46] had a low risk of bias in random sequence generation, indicating the use of appropriate randomization methods. However, approximately 50% (13/24) of the studies [25,28,31,33,34,36,39,40,43-46,48] had unclear allocation concealment, and over 10% (3/24) of the studies [30,38,47] had a high risk, suggesting potential issues with randomization. In contrast, 75% (18/24) of the studies [26-28,30-33,37-43,45-48] had a low risk of bias in blinding of outcome assessors. In addition, 95% (23/24) of the studies [25-45,47,48] demonstrated a low risk of bias related to incomplete outcome data, though a small number had an unclear risk. Over 80% (20/24) of the studies [25,26,28-37,40-46,48] showed a low risk of selective reporting, with no evidence of reporting bias. Overall, the risk of other biases was minimal. In conclusion, while concerns remain regarding allocation concealment and blinding, approximately 70% (17/24) of the studies [25,26,29,31-37,40-43,45,46,48] had a low risk of bias, supporting the credibility of the meta-analysis findings (Figures8  9). The funnel plot (Multimedia Appendix 5) showed a symmetrical distribution, with most studies clustered at the top, suggesting potential small-sample effects, as negative or nonsignificant findings are less likely to be published. The Egger test did not indicate the presence of publication bias (*P*>.05). The funnel plots are shown in agreement with statistical tests because they lack significant asymmetry.

**Figure 9.**
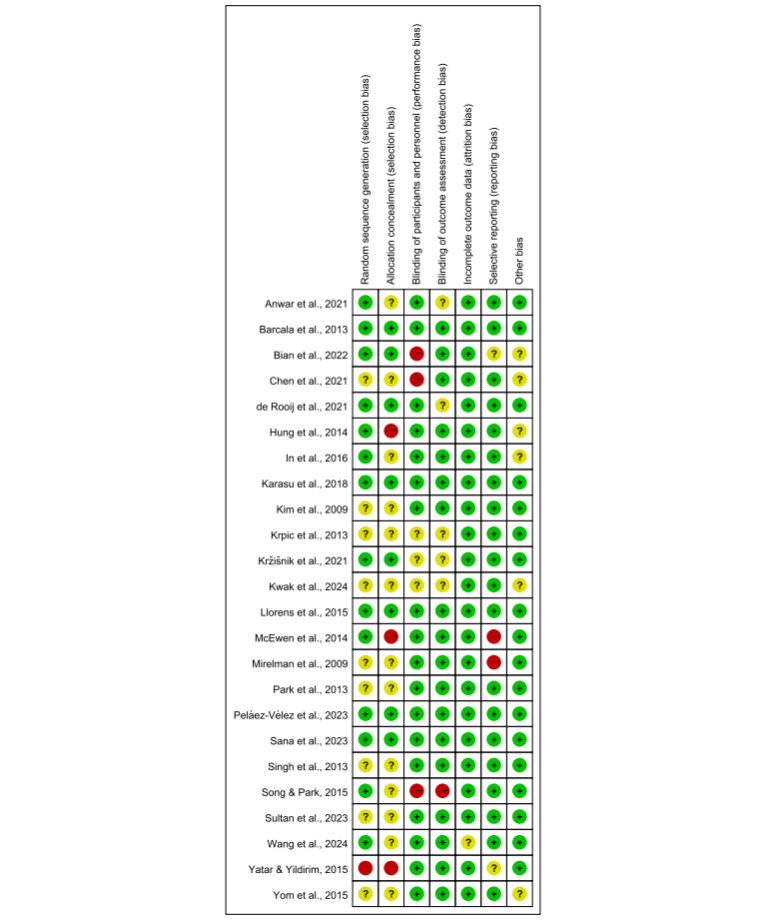
Risk of bias summary [25-48].

## Discussion

### Principal Findings

This study included 24 RCTs [25-48] with a total of 768 participants. Our findings support the initial hypothesis that VR-based interventions significantly improve lower limb function compared to conventional therapy. The observed positive effects on lower limb rehabilitation, particularly in BBS, TUG, and step length, underscore the potential of VR therapy in advancing rehabilitation approaches and improving patient outcomes. VR technology has proven effective in facilitating motor learning through multisensory integration and task-oriented training [49,50]. VR environments provide real-time visual, auditory, and proprioceptive feedback, which can enhance neuroplasticity and promote motor function recovery [51]. The immersive multisensory interaction in VR also improves patient attention, motivation, and engagement during rehabilitation [52]. In addition, task-oriented training in VR allows patients to engage in repetitive, goal-directed movements that mimic real-life activities, reinforcing motor control and improving balance and gait performance [53]. This approach motivates patients to practice essential movements repetitively, leading to improved outcomes. These mechanisms collectively highlight the potential of VR in enhancing rehabilitation outcomes, providing a foundation for further analysis in the subsequent subgroup results. Subgroup analyses showed that VR therapy was more effective in patients more than 6 months after stroke and less effective between 7 days and 6 months after stroke in BBS. According to recommendations from the Mayo Clinic [54], stroke rehabilitation typically begins within 24-48 hours after stroke onset, with the highest rates of recovery generally occurring within weeks and months following the stroke. However, our study found that VR therapy demonstrated statistically different effectiveness in patients with stroke more than 6 months after stroke. This seems to represent a potential rehabilitation benefit from VR therapy even in patients who have had a stroke more than 6 months after the stroke. Regarding the impact of different VR types on outcomes, studies have shown that NIVR was effective in improving TUG test results in patients with stroke, whereas SIVR and IVR were not statistically different. Most importantly, our study demonstrated that for stroke survivors, 20 or more sessions of VR treatment resulted in statistically significant improvements in balance and mobility, while less than 20 sessions did not. Therefore, this study suggests that higher treatment frequency, especially more than 20 sessions, is more helpful in improving balance and mobility functions. A study [55] conducted a subgroup analysis on the effect of VR on upper limb function recovery in patients with stroke based on intervention frequency, which found that when VR intervention frequency exceeded 18 sessions, upper limb motor function outcomes showed statistical differences compared to fewer than 18 sessions. This is similar to our study, where lower limb functions (BBS and TUG) began to show statistical differences when the total intervention frequency reached ≥20 sessions.

### Heterogeneity

Regarding the high heterogeneity of BBS results, we have conducted subgroup analyses based on the time after stroke, VR type, and total frequency. The results showed that time after stroke and differences within the NIVR group may be the cause of heterogeneity. At the same time, we found that the heterogeneity of the group with a total frequency greater than or equal to 20 times was high, which may be due to the different durations and weekly times of different study schemes. In addition, we sorted out VR devices and intervention content (Multimedia Appendix 2) and conducted a sensitivity analysis (Multimedia Appendix 3). The results showed that the main source of heterogeneity came from studies using Nintendo Wii, with a value as high as 90%. The intervention content differed in each study, which prevented us from performing a subgroup analysis by intervention content. We added the intervention content and VR devices of each study in the table named “Baseline Characteristics of Included Studies” (Multimedia Appendix 2). In addition, we conducted a sensitivity analysis by the leave-one-out method and found that one study [25] was an important source of heterogeneity, ie, the overall heterogeneity of the studies changed from 81% to 35% after removing this study, but the overall heterogeneity remained 78%‐83% after removing the other studies in turn, but unfortunately, we did not find a significant source of heterogeneity for this study.

### Implications for Clinical Practice

Currently, the American Stroke Association’s recommended rehabilitation measures for poststroke recovery include physical therapy, audiology, recreational therapy, and rehabilitation nursing. As a form of audiology recreational therapy, VR treatment can create immersive VR environments that generate an illusion of bodily movement. This immersive experience can increase the activation of brain areas related to movement, thereby boosting neuroplasticity and facilitating the reorganization of synaptic connections within the nervous system. By impacting the central nervous system directly, VR therapy plays a key role in reshaping and aiding the recovery of neural structures after a stroke, contributing significantly to rehabilitation and the restoration of motor functions [56]. When considering VR as a vital component of stroke rehabilitation, this study points out that the following four factors should be taken into account:

1. Patient’s medical condition, including the type of prior stroke, stroke severity, number of strokes, and any mental health conditions such as depression or anxiety poststroke.
2. Patient’s background, including economic status, education level, and geographical location. A study [57] pointed out that individuals with different economic and educational backgrounds may have varying levels of access to and familiarity with VR technology, which could potentially affect the efficacy of VR therapy.
3. Specific interventions of VR therapy, including the type of VR, type of games, duration of VR intervention, frequency of VR use per week, and total number of sessions.
4. Outcome assessment: in addition to lower limb function as examined in this study, 45 assessments of upper limb function, cognitive function, and hand function are also important aspects to consider.

### Strengths and Limitations

A key limitation of this study is the relatively small sample size due to the lack of large-scale population studies, which may affect the statistical power and generalizability of the results. The study also assumed that data were normally distributed, and the conversion of medians to means in some studies may introduce errors [58], especially if the data deviate from normality. Furthermore, the calculation of changes in SD assumed a correlation coefficient (*r*) of 0.5, without conducting sensitivity analyses to explore the impact of different *r* values, which could affect the accuracy of SD calculations. In addition, the study did not evaluate the safety of VR therapy, and although it is generally considered safe, some studies report minor adverse effects like dizziness or headaches [59]. Besides, since the patients in the included studies did not differentiate whether the patients had a previous hemorrhagic stroke or ischemic stroke, one of the limitations of our study was that we could not further give separate personalized VR protocols. Finally, our limitation is the high heterogeneity of our study, the source of which may be due to the fact that the interventions in the control group were not uniform, for example, balance training in some studies and treadmill exercise in others. Therefore, we addressed this issue through subgroup analysis and sensitivity analysis. Future research should address these limitations by using larger sample sizes, obtaining original mean and SD data, verifying assumptions on correlation, and systematically evaluating the safety of VR-based therapy.

A strength of our study is the inclusion of a larger number of studies in the meta-analysis. This increased sample size allowed for more extensive subgroup analyses compared to previous studies, which provided valuable insights into factors influencing the effectiveness of VR-based therapy. In addition, our study revealed that higher intervention frequency may play a crucial role in stroke rehabilitation, offering guidance for future treatment strategies.

### Conclusion

Meta-analysis of RCTs demonstrates that VR therapy is effective in improving lower limb function in stroke survivors, with higher treatment frequency (particularly ≥20 sessions) yielding superior outcomes. These findings highlight the importance of session intensity in maximizing rehabilitation benefits and provide strong evidence to support the integration of VR-based therapies into standard rehabilitation protocols for patients with stroke. Compared to earlier studies, this review emphasizes the critical role of treatment frequency, demonstrating that increased VR therapy sessions significantly enhance recovery, particularly in balance and functional mobility. Based on these results, we recommend that ≥20 sessions of VR interventions be incorporated into clinical guidelines for chronic stroke rehabilitation to optimize functional outcomes. Furthermore, the accessibility and cost-effectiveness of VR interventions should be considered by policy makers for potential inclusion in medical insurance coverage, which could improve patient access and reduce long-term health care costs. However, limitations such as the relatively small sample size and the need for more comprehensive safety evaluations highlight the necessity for further investigation. Future research should examine the effects of various VR technologies and environments on stroke recovery to refine therapeutic strategies and enhance clinical outcomes.

## Supplementary material

10.2196/72364 Multimedia Appendix 1 Search strategy.

10.2196/72364 Multimedia Appendix 2 Characteristics of included studies.

10.2196/72364 Multimedia Appendix 3 Sensitivity analysis of VR effects on balance (measured by BBS), based on different VR devices. VR: virtual reality; BBS: Berg Balance Scale.

10.2196/72364 Multimedia Appendix 4 Forest plots for virtual reality on subgroup, stride length, Functional Reach Test, Dynamic Gait Index, and Falls Efficacy Scale-International.

10.2196/72364 Multimedia Appendix 5 Funnel plot of publication bias.

10.2196/72364 Checklist 1 Preferred Reporting Items for Systematic Reviews and Meta-Analysis checklist.

## Abbreviations

10-MWT: 10-Meter Walk Test
BBS: Berg Balance Scale
CENTRAL: Cochrane Central Register of Controlled Trials
DGI: Dynamic Gait Index
FES-I: Falls Efficacy Scale-International
FRT: Functional Reach Test
INPLASY: International Platform of Registered Systematic Review and Meta-Analysis Protocols
IVR: immersive virtual reality
MD: mean difference
NIVR: nonimmersive virtual reality
PRISMA: Preferred Reporting Items for Systematic Reviews and Meta-Analysis
RCT: randomized controlled trial
RoB: Risk of Bias
SIVR: semi-immersive virtual reality
TUG: Timed Up and Go Test
VR: virtual reality
