# Supplementary material for: Effect of Virtual Reality–Based Therapies on Lower Limb Functional Recovery in Stroke Survivors: Systematic Review and Meta-Analysis
Source: J Med Internet Res. 2025 Jul 30;27:e72364. doi: 10.2196/72364 (PMC12310189; doi:10.2196/72364)
Supplement: Multimedia Appendix 1 [file jmir-v27-e72364-s001.docx]

**APPENDIX 1**

**Search strategy**

| **Medline (Ovid)** | | |
| --- | --- | --- |
| **S.no** | **Topic** | **Hits** |
| **1** | exp Virtual Reality/ | 7081 |
| **2** | (virtual realit* or virtual realit* thera*).mp. [mp=title, book title, abstract, original title, name of substance word, subject heading word, floating sub-heading word, keyword heading word, organism supplementary concept word, protocol supplementary concept word, rare disease supplementary concept word, unique identifier, synonyms, population supplementary concept word, anatomy supplementary concept word] | 21488 |
| **3** | VR.mp. [mp=title, book title, abstract, original title, name of substance word, subject heading word, floating sub-heading word, keyword heading word, organism supplementary concept word, protocol supplementary concept word, rare disease supplementary concept word, unique identifier, synonyms, population supplementary concept word, anatomy supplementary concept word] | 14606 |
| **4** | (comput* adj3 simulat*).mp. [mp=title, book title, abstract, original title, name of substance word, subject heading word, floating sub-heading word, keyword heading word, organism supplementary concept word, protocol supplementary concept word, rare disease supplementary concept word, unique identifier, synonyms, population supplementary concept word, anatomy supplementary concept word] | 244268 |
| **5** | 1 or 2 or 3 or 4 | 269144 |
| **6** | exp stroke/ or exp rehabilitation/ or exp neurological rehabilitation/ or exp stroke rehabilitation/ | 530999 |
| **7** | (stroke* or CVA or apoplex* or cerebral* hemorrhag* or brain vascular accident or stroke survivor* or post-stroke or poststroke or stroke patient* or stroke rehab* or stroke recover* or stroke thera*).mp. [mp=title, book title, abstract, original title, name of substance word, subject heading word, floating sub-heading word, keyword heading word, organism supplementary concept word, protocol supplementary concept word, rare disease supplementary concept word, unique identifier, synonyms, population supplementary concept word, anatomy supplementary concept word] | 443412 |
| **8** | 6 or 7 | 803719 |
| **9** | exp Lower Extremity/ | 191943 |
| **10** | (low* limb* or lower leg* or membrum inferius*).mp. [mp=title, book title, abstract, original title, name of substance word, subject heading word, floating sub-heading word, keyword heading word, organism supplementary concept word, protocol supplementary concept word, rare disease supplementary concept word, unique identifier, synonyms, population supplementary concept word, anatomy supplementary concept word] | 79097 |
| **11** | 9 or 10 | 245124 |
| **12** | exp Postural Balance/ | 29343 |
| **13** | (balance or balance* abilit* or balance* or (postur* adj1 (control or stability or instability)) or equilibrium*).mp. [mp=title, book title, abstract, original title, name of substance word, subject heading word, floating sub-heading word, keyword heading word, organism supplementary concept word, protocol supplementary concept word, rare disease supplementary concept word, unique identifier, synonyms, population supplementary concept word, anatomy supplementary concept word] (590529) | 590529 |
| **14** | 12 or 13 | 590554 |
| **15** | exp gait/ or exp gait analysis/ or exp walking speed/ | 39240 |
| **16** | (gait* or gait analys* or (gait adj2 analys*) or gait speed).mp. [mp=title, book title, abstract, original title, name of substance word, subject heading word, floating sub-heading word, keyword heading word, organism supplementary concept word, protocol supplementary concept word, rare disease supplementary concept word, unique identifier, synonyms, population supplementary concept word, anatomy supplementary concept word] | 83172 |
| **17** | (walk* speed or walk* pace or walk* abilit*).mp. [mp=title, book title, abstract, original title, name of substance word, subject heading word, floating sub-heading word, keyword heading word, organism supplementary concept word, protocol supplementary concept word, rare disease supplementary concept word, unique identifier, synonyms, population supplementary concept word, anatomy supplementary concept word] | 14824 |
| **18** | 15 or 16 or 17 | 90640 |
| **19** | 11 or 14 or 18 | 886320 |
| **20** | exp randomized controlled trial/ | 619572 |
| **21** | controlled clinical trial.pt. | 95579 |
| **22** | randomized.ab. | 654816 |
| **23** | placebo.ab. | 250470 |
| **24** | drug therapy.fs. | 2718065 |
| **25** | randomly.ab. | 438863 |
| **26** | trial.ab. | 708250 |
| **27** | groups.ab. | 2713524 |
| **28** | 20 or 21 or 22 or 23 or 24 or 25 or 26 or 27 | 6026250 |
| **29** | exp animals/ not humans.sh. | 5243854 |
| **30** | 28 not 29 | 5274381 |
| **31** | 5 and 8 and 19 and 30 | **503** |

| **Embase (Ovid)** | | |
| --- | --- | --- |
| **S.no** | **Topic** | **Hits** |
| **1** | exp Virtual Reality/ | 29913 |
| **2** | (virtual realit* or virtual realit* thera*).mp. [mp=title, abstract, heading word, drug trade name, original title, device manufacturer, drug manufacturer, device trade name, keyword heading word, floating subheading word, candidate term word] | 37188 |
| **3** | VR.mp. [mp=title, abstract, heading word, drug trade name, original title, device manufacturer, drug manufacturer, device trade name, keyword heading word, floating subheading word, candidate term word] | 20798 |
| **4** | (comput* adj3 simulat*).mp. [mp=title, abstract, heading word, drug trade name, original title, device manufacturer, drug manufacturer, device trade name, keyword heading word, floating subheading word, candidate term word] | 167202 |
| **5** | 1 or 2 or 3 or 4 | 212062 |
| **6** | exp stroke/ or exp rehabilitation/ or exp neurological rehabilitation/ or exp stroke rehabilitation/ | 838619 |
| **7** | (stroke* or CVA or apoplex* or cerebral* hemorrhag* or brain vascular accident or stroke survivor* or post-stroke or poststroke or stroke patient* or stroke rehab* or stroke recover* or stroke thera*).mp. [mp=title, abstract, heading word, drug trade name, original title, device manufacturer, drug manufacturer, device trade name, keyword heading word, floating subheading word, candidate term word] | 629471 |
| **8** | 6 or 7 | 1180160 |
| **9** | exp Lower Extremity/ | 522199 |
| **10** | (low* limb* or lower leg* or membrum inferius*).mp. [mp=title, abstract, heading word, drug trade name, original title, device manufacturer, drug manufacturer, device trade name, keyword heading word, floating subheading word, candidate term word] | 143948 |
| **11** | 9 or 10 | 577967 |
| **12** | exp Postural Balance/ | 24719 |
| **13** | (balance or balance* abilit* or balance* or (postur* adj1 (control or stability or instability)) or equilibrium*).mp. [mp=title, abstract, heading word, drug trade name, original title, device manufacturer, drug manufacturer, device trade name, keyword heading word, floating subheading word, candidate term word] | 721682 |
| **14** | 12 or 13 | 721842 |
| **15** | exp gait/ or exp gait analysis/ or exp walking speed/ | 89118 |
| **16** | (gait* or gait analys* or (gait adj2 analys*) or gait speed).mp. [mp=title, abstract, heading word, drug trade name, original title, device manufacturer, drug manufacturer, device trade name, keyword heading word, floating subheading word, candidate term word] | 135728 |
| **17** | (walk* speed or walk* pace or walk* abilit*).mp. [mp=title, abstract, heading word, drug trade name, original title, device manufacturer, drug manufacturer, device trade name, keyword heading word, floating subheading word, candidate term word] | 34136 |
| **18** | 15 or 16 or 17 | 150435 |
| **19** | 11 or 14 or 18 | 1367675 |
| **20** | exp randomized controlled trial/ | 839268 |
| **21** | controlled clinical trial/ | 473679 |
| **22** | random$.ti,ab. | 2104695 |
| **23** | randomization/ | 99825 |
| **24** | intermethod comparison/ | 307861 |
| **25** | placebo.ti,ab. | 381571 |
| **26** | (compare or compared or comparison).ti,ab. | 8078750 |
| **27** | ((evaluated or evaluate or evaluating or assessed or assess) and (compare or compared or comparing or comparison)).mp. [mp=title, abstract, heading word, drug trade name, original title, device manufacturer, drug manufacturer, device trade name, keyword heading word, floating subheading word, candidate term word] | 3193222 |
| **28** | (open adj label).ti,ab. | 117351 |
| **29** | ((double or single or doubly or singly) adj (blind or blinded or blindly)).ti,ab. | 285763 |
| **30** | double blind procedure/ | 222283 |
| **31** | parallel group$1.ti,ab. | 34070 |
| **32** | (crossover or cross over).ti,ab. | 129833 |
| **33** | ((assign$ or match or matched or allocation) adj5 (alternate or group$1 or intervention$1 or patient$1 or subject$1 or participant$1)).ti,ab. | 439786 |
| **34** | (assigned or allocated).ti,ab. | 519949 |
| **35** | (controlled adj7 (study or design or trial)).ti,ab. | 480283 |
| **36** | (volunteer or volunteers).ti,ab. | 291603 |
| **37** | human experiment/ | 667899 |
| **38** | trial.ti. | 433044 |
| **39** | or/20-38 | 10689913 |
| **40** | (random$ adj sampl$ adj7 ("cross section$" or questionnaire$1 or survey$ or database$1)).ti,ab. not (comparative study/ or controlled study/ or randomi?ed controlled.ti,ab. or randomly assigned.ti,ab.) | 10114 |
| **41** | cross-sectional study/de not (exp randomized controlled trial/ or controlled clinical trial/ or controlled study/ or randomi?ed controlled.ti,ab. or control group$1.ti,ab.) | 0 |
| **42** | ((case adj control$).mp. and random$.ti,ab.) not randomi?ed controlled.ti,ab. [mp=title, abstract, heading word, drug trade name, original title, device manufacturer, drug manufacturer, device trade name, keyword heading word, floating subheading word, candidate term word] | 27664 |
| **43** | (nonrandom$ not random$).ti,ab. | 19725 |
| **44** | "random field$".ti,ab. | 3097 |
| **45** | (random cluster adj3 sampl$).ti,ab. | 1672 |
| **46** | (review.ab. and review.pt.) not trial.ti. | 1220594 |
| **47** | (rat or rats or mouse or mice or swine or porcine or murine or sheep or lambs or pigs or piglets or rabbit or rabbits or cat or cats or dog or dogs or cattle or bovine or monkey or monkeys or trout or marmoset$1).ti. and animal experiment/ | 1263915 |
| **48** | animal experiment/ not (human experiment/ or human/) | 2658037 |
| **49** | systematic review.ti,ab. | 374397 |
| **50** | (review.ab. and review.pt.) not trial.ti. | 1220594 |
| **51** | "we searched".ab. and (review.ti. or review.pt.) | 54355 |
| **52** | "update review".ab. | 144 |
| **53** | (databases adj4 searched).ab. | 70500 |
| **54** | or/40-53 | 4261841 |
| **55** | 39 not 54 | 9429982 |
| **56** | 5 and 8 and 19 and 55 | **643** |

| **PubMed** | | |
| --- | --- | --- |
| **S.no** | **Topic** | **Hits** |
| #1 | "Virtual Reality"[MeSH Terms] | 7,053 |
| #2 | "virtual reality"[Title/Abstract] OR "virtual reality exposure therapy"[Title/Abstract] OR "VR"[Title/Abstract] OR "vr intervention"[Title/Abstract] OR "game based virtual reality"[Title/Abstract] OR "computer based virtual reality"[Title/Abstract] OR "computer simulation"[Title/Abstract] OR "virtual reality exercises"[Title/Abstract] | 40,915 |
| #3 | "Virtual Reality"[MeSH Terms] OR "Virtual Reality"[Title/Abstract] OR "virtual reality exposure therapy"[Title/Abstract] OR "VR"[Title/Abstract] OR "vr intervention"[Title/Abstract] OR "game based virtual reality"[Title/Abstract] OR "computer based virtual reality"[Title/Abstract] OR "computer simulation"[Title/Abstract] OR "virtual reality exercises"[Title/Abstract] | 42,132 |
| #4 | "Stroke"[MeSH Terms] | 183,305 |
| #5 | "Stroke"[Title/Abstract] OR "cerebral vascular accident"[Title/Abstract] OR "hemiplegia"[Title/Abstract] OR "cerebral stroke"[Title/Abstract] OR "brain vascular accident"[Title/Abstract] OR "Apoplexy"[Title/Abstract] OR "CVA"[Title/Abstract] OR "poststroke"[Title/Abstract] OR "post-stroke"[Title/Abstract] OR "stroke survivor"[Title/Abstract] | 348,096 |
| #6 | "Stroke"[MeSH Terms] OR "Stroke"[Title/Abstract] OR "cerebral vascular accident"[Title/Abstract] OR "hemiplegia"[Title/Abstract] OR "cerebral stroke"[Title/Abstract] OR "brain vascular accident"[Title/Abstract] OR "Apoplexy"[Title/Abstract] OR "CVA"[Title/Abstract] OR "poststroke"[Title/Abstract] OR "post-stroke"[Title/Abstract] OR "stroke survivor"[Title/Abstract] | 388,123 |
| #7 | "Lower Extremity"[MeSH Terms] | 191,931 |
| #8 | "lower limb"[Title/Abstract] OR "lower leg"[Title/Abstract] OR "motor function"[Title/Abstract] OR "lower limb function"[Title/Abstract] OR "lower extremities"[Title/Abstract] OR "membrum inferius"[Title/Abstract] OR "Balance"[Title/Abstract] OR "posture balance"[Title/Abstract] OR "balance control"[Title/Abstract] OR "postural equilibrium"[Title/Abstract] OR "gait"[Title/Abstract] OR "gait analysis"[Title/Abstract] OR "gait disorder"[Title/Abstract] OR "walking speed"[Title/Abstract] OR "walking pace"[Title/Abstract] OR "activity of daily living"[Title/Abstract] OR "ADL"[Title/Abstract] | 478,670 |
| #9 | "Lower Extremity"[MeSH Terms] OR "lower limb"[Title/Abstract] OR "lower leg"[Title/Abstract] OR "motor function"[Title/Abstract] OR "lower limb function"[Title/Abstract] OR "lower extremities"[Title/Abstract] OR "membrum inferius"[Title/Abstract] OR "Balance"[Title/Abstract] OR "posture balance"[Title/Abstract] OR "balance control"[Title/Abstract] OR "postural equilibrium"[Title/Abstract] OR "gait"[Title/Abstract] OR "gait analysis"[Title/Abstract] OR "gait disorder"[Title/Abstract] OR "walking speed"[Title/Abstract] OR "walking pace"[Title/Abstract] OR "activity of daily living"[Title/Abstract] OR "ADL"[Title/Abstract] | 631,523 |
| #10 | "randomized controlled trial"[Publication Type] OR "controlled clinical trial"[Publication Type] OR "clinical trials as topic"[MeSH Terms:noexp] OR "trial"[Title] OR "random*"[Title/Abstract] OR "placebo*"[Title/Abstract] | 2,021,722 |
| #11 | ("Virtual Reality"[MeSH Terms] OR ("Virtual Reality"[Title/Abstract] OR "virtual reality exposure therapy"[Title/Abstract] OR ("VR"[Title/Abstract] OR "vr intervention"[Title/Abstract]) OR "game based virtual reality"[Title/Abstract] OR "computer based virtual reality"[Title/Abstract] OR "computer simulation"[Title/Abstract] OR "virtual reality exercises"[Title/Abstract])) AND ("Stroke"[MeSH Terms] OR ("Stroke"[Title/Abstract] OR "cerebral vascular accident"[Title/Abstract] OR "hemiplegia"[Title/Abstract] OR "cerebral stroke"[Title/Abstract] OR "brain vascular accident"[Title/Abstract] OR "Apoplexy"[Title/Abstract] OR "CVA"[Title/Abstract] OR ("poststroke"[Title/Abstract] OR "post-stroke"[Title/Abstract] OR "stroke survivor"[Title/Abstract]))) AND ("Lower Extremity"[MeSH Terms] OR ("lower limb"[Title/Abstract] OR "lower leg"[Title/Abstract] OR ("motor function"[Title/Abstract] OR "lower limb function"[Title/Abstract]) OR "lower extremities"[Title/Abstract] OR "membrum inferius"[Title/Abstract] OR "Balance"[Title/Abstract] OR "posture balance"[Title/Abstract] OR "balance control"[Title/Abstract] OR "postural equilibrium"[Title/Abstract] OR "gait"[Title/Abstract] OR "gait analysis"[Title/Abstract] OR "gait disorder"[Title/Abstract] OR ("walking speed"[Title/Abstract] OR "walking pace"[Title/Abstract]) OR "activity of daily living"[Title/Abstract] OR "ADL"[Title/Abstract])) AND ("randomized controlled trial"[Publication Type] OR "controlled clinical trial"[Publication Type] OR "clinical trials as topic"[MeSH Terms:noexp] OR "trial"[Title] OR "random*"[Title/Abstract] OR "placebo*"[Title/Abstract]) | 274 |
| #12 | (("Virtual Reality"[MeSH Terms] OR ("Virtual Reality"[Title/Abstract] OR "virtual reality exposure therapy"[Title/Abstract] OR ("VR"[Title/Abstract] OR "vr intervention"[Title/Abstract]) OR "game based virtual reality"[Title/Abstract] OR "computer based virtual reality"[Title/Abstract] OR "computer simulation"[Title/Abstract] OR "virtual reality exercises"[Title/Abstract])) AND ("Stroke"[MeSH Terms] OR ("Stroke"[Title/Abstract] OR "cerebral vascular accident"[Title/Abstract] OR "hemiplegia"[Title/Abstract] OR "cerebral stroke"[Title/Abstract] OR "brain vascular accident"[Title/Abstract] OR "Apoplexy"[Title/Abstract] OR "CVA"[Title/Abstract] OR ("poststroke"[Title/Abstract] OR "post-stroke"[Title/Abstract] OR "stroke survivor"[Title/Abstract]))) AND ("Lower Extremity"[MeSH Terms] OR ("lower limb"[Title/Abstract] OR "lower leg"[Title/Abstract] OR ("motor function"[Title/Abstract] OR "lower limb function"[Title/Abstract]) OR "lower extremities"[Title/Abstract] OR "membrum inferius"[Title/Abstract] OR "Balance"[Title/Abstract] OR "posture balance"[Title/Abstract] OR "balance control"[Title/Abstract] OR "postural equilibrium"[Title/Abstract] OR "gait"[Title/Abstract] OR "gait analysis"[Title/Abstract] OR "gait disorder"[Title/Abstract] OR ("walking speed"[Title/Abstract] OR "walking pace"[Title/Abstract]) OR "activity of daily living"[Title/Abstract] OR "ADL"[Title/Abstract])) AND ("randomized controlled trial"[Publication Type] OR "controlled clinical trial"[Publication Type] OR "clinical trials as topic"[MeSH Terms:noexp] OR "trial"[Title] OR "random*"[Title/Abstract] OR "placebo*"[Title/Abstract])) AND (randomizedcontrolledtrial[Filter]) | **100** |

| **Cochrane(CENTRAL)** | | |
| --- | --- | --- |
| **S.no** | **Topic** | **Hits** |
| #1 | MeSH descriptor: [Stroke] explode all trees | 17550 |
| #2 | (Strokes):ti,ab,kw OR (Cerebrovascular Accident):ti,ab,kw OR (Cerebrovascular Accidents):ti,ab,kw OR (CVA):ti,ab,kw OR (CVAs):ti,ab,kw | 21750 |
| #3 | (Apoplexy, Cerebrovascular):ti,ab,kw OR (Cerebrovascular Apoplexy):ti,ab,kw OR (Vascular Accident, Brain):ti,ab,kw OR (Brain Vascular Accident*):ti,ab,kw (Word variations have been searched) | 605 |
| #4 | (Vascular Accidents, Brain):ti,ab,kw OR (Cerebrovascular Stroke*):ti,ab,kw OR (Stroke, Cerebrovascular):ti,ab,kw OR (Strokes, Cerebrovascular):ti,ab,kw OR (Apoplexy):ti,ab,kw (Word variations have been searched) | 19388 |
| #5 | (Cerebral Stroke):ti,ab,kw OR (Cerebral Strokes):ti,ab,kw OR (Stroke, Cerebral):ti,ab,kw OR (Strokes, Cerebral):ti,ab,kw (Word variations have been searched) | 8490 |
| #6 | (Stroke, Acute):ti,ab,kw OR (Acute Stroke):ti,ab,kw OR (Acute Strokes):ti,ab,kw OR (Strokes, Acute):ti,ab,kw (Word variations have been searched) | 20060 |
| #7 | (Cerebrovascular Accident, Acute):ti,ab,kw OR (Acute Cerebrovascular Accident):ti,ab,kw OR (Acute Cerebrovascular Accidents):ti,ab,kw OR (Cerebrovascular Accidents, Acute):ti,ab,kw (Word variations have been searched) | 4846 |
| #8 | #2 OR #3 OR #4 OR #5 OR #6 OR #7 | 42237 |
| #9 | #1 OR #8 | 49449 |
| #10 | MeSH descriptor: [Virtual Reality] explode all trees | 1156 |
| #11 | ("virtual reality therapy"):ti,ab,kw OR ("VR therapy"):ti,ab,kw OR (virtual reality exposure therapy):ti,ab,kw (Word variations have been searched) | 987 |
| #12 | (computer simulation):ti,ab,kw AND (video NEXT game*):ti,ab,kw (Word variations have been searched) | 100 |
| #13 | #11 OR #12 | 1082 |
| #14 | #10 OR #13 | 2120 |
| #15 | MeSH descriptor: [Upper Extremity] explode all trees | 10711 |
| #16 | (lower limb*):ti,ab,kw OR (lower?limb muscle*):ti,ab,kw OR (lower extremit*):ti,ab,kw (Word variations have been searched) | 27232 |
| #17 | #15 OR #16 | 37316 |
| #18 | MeSH descriptor: [Postural Balance] explode all trees | 4324 |
| #19 | (postur* NEXT control):ti,ab,kw OR (postur* NEXT stability):ti,ab,kw OR (Balance, Posture):ti,ab,kw (Word variations have been searched) | 9018 |
| #20 | #18 OR #19 | 9034 |
| #21 | MeSH descriptor: [Gait] explode all trees | 3296 |
| #22 | (gait analy*):ti,ab,kw OR (gait disorder):ti,ab,kw OR (gait or locomot* or ambulat* or walk* or mobility):ti,ab,kw OR (walk* NEXT speed or pace):ti,ab,kw (Word variations have been searched) | 123006 |
| #23 | #21 OR #22 | 123006 |
| #24 | MeSH descriptor: [Activities of Daily Living] explode all trees | 13271 |
| #25 | (ADL):ti,ab,kw OR (daily liv* activit*):ti,ab,kw (Word variations have been searched) | 22841 |
| #26 | #24 OR #25 | 29085 |
| #27 | #17 OR #20 OR #23 OR #26 | 176650 |
| #28 | #9 AND #14 AND #27 | **142** |

| **Web of Science (WOS)** | | |
| --- | --- | --- |
| **S.no** | **Topic** | **Hits** |
| #1 | TI=((((Stroke*) OR (CVA) OR (Cerebrovas*) OR (Poststroke*) OR (post-stroke*) OR (Hemipleg*) OR (paretic) OR (apoplexy) OR (paresis) OR (Hemipare*) OR (Neuroplast*)) OR (((cerebral*) OR (Brain*) OR (cerebellar)) AND ((infarct*) OR (isch?mi*) OR (thrombo*) OR (emboli*) OR (apoplex*) OR (attack*) OR (vascular*) OR (injur*))) OR (((cerebral) OR (Brain) OR (cerebella)) AND ((haemorrhage) OR (hemorrhage) OR (haematoma) OR (hematoma) OR (bleed*))) OR (“Transient ischemic attack”) OR (TIA) OR (“Subarachnoid hemorrhage”) OR (“Gait Disorders”))) | 379,351 |
| #2 | TS=(virtual reality OR "virtual reality" OR virtual* realit* OR "VR" OR "Video* game*" OR "Serious* game*" OR Comput* assist* OR comput* base* OR immers* OR "semi* immers*" OR Gamificat*) | 1,934,269 |
| #3 | TS=(Rehab* OR recovery OR restore* OR Habilitat* OR therapy OR treat* OR care OR plan* OR intervent* OR approach* OR interact* OR Counsel* or Heal* OR aid* OR help* OR relief* OR well* OR support OR program OR software OR system OR device OR technique*) | 42,026,947 |
| #4 | #2 AND #3 | 1,593,258 |
| #5 | TS=("Clinical Trial*" OR "Random* Control* Trial*" OR "RCT") | 928,097 |
| #6 | #1 AND #4 AND #5 | 1,218 |
| #7 | TS=(upper limb or upper extremity or upper* limb* or upper* extrem*) | 119,426 |
| #8 | #6 NOT #7 | 853 |
| #9 | #6 NOT #7 and Article (Document Types) | **644** |

**APA PsycInfo (Ovid)**

**1**  exp Virtual Reality/ (13365)
**2**  (virtual realit* or virtual realit* thera*).mp. [mp=title, abstract, heading word, table of contents, key concepts, original title, tests & measures, mesh word] (15143)
**3**  VR.mp. [mp=title, abstract, heading word, table of contents, key concepts, original title, tests & measures, mesh word] (6017)
**4**  (comput* adj3 simulat*).mp. [mp=title, abstract, heading word, table of contents, key concepts, original title, tests & measures, mesh word] (17611)
**5**  1 or 2 or 3 or 4 (34508)
**6**  exp stroke/ or exp rehabilitation/ or exp neurological rehabilitation/ or exp stroke rehabilitation/ (84638)
**7**  (stroke* or CVA or apoplex* or cerebral* hemorrhag* or brain vascular accident or stroke survivor* or post-stroke or poststroke or stroke patient* or stroke rehab* or stroke recover* or stroke thera*).mp. [mp=title, abstract, heading word, table of contents, key concepts, original title, tests & measures, mesh word] (46903)
**8**  6 or 7 (105472)
**9**  exp Lower Extremity/ (0)
**10**  (low* limb* or lower leg* or membrum inferius*).mp. [mp=title, abstract, heading word, table of contents, key concepts, original title, tests & measures, mesh word] (4163)
**11**  9 or 10 (4163)
**12**  exp Postural Balance/ (0)
**13**  (balance or balance* abilit* or balance* or (postur* adj1 (control or stability or instability)) or equilibrium*).mp. [mp=title, abstract, heading word, table of contents, key concepts, original title, tests & measures, mesh word] (92341)
**14**  12 or 13 (92341)
**15**  exp gait/ or exp gait analysis/ or exp walking speed/ (4508)
**16**  (gait* or gait analys* or (gait adj2 analys*) or gait speed).mp. [mp=title, abstract, heading word, table of contents, key concepts, original title, tests & measures, mesh word] (12672)
**17**  (walk* speed or walk* pace or walk* abilit*).mp. [mp=title, abstract, heading word, table of contents, key concepts, original title, tests & measures, mesh word] (2145)
**18**  15 or 16 or 17 (13881)
**19**  11 or 14 or 18 (105630)
**20**  exp randomized controlled trial/ (1642)
**21**  controlled clinical trial/ (0)
**22**  random$.ti,ab. (254297)
**23**  randomization/ (0)
**24**  intermethod comparison/ (0)
**25**  placebo.ti,ab. (44782)
**26**  (compare or compared or comparison).ti,ab. (800094)
**27**  ((evaluated or evaluate or evaluating or assessed or assess) and (compare or compared or comparing or comparison)).mp. [mp=title, abstract, heading word, table of contents, key concepts, original title, tests & measures, mesh word] (225516)
**28**  (open adj label).ti,ab. (7234)
**29**  ((double or single or doubly or singly) adj (blind or blinded or blindly)).ti,ab. (29679)
**30**  double blind procedure/ (0)
**31**  parallel group$1.ti,ab. (3124)
**32**  (crossover or cross over).ti,ab. (12237)
**33**  ((assign$ or match or matched or allocation) adj5 (alternate or group$1 or intervention$1 or patient$1 or subject$1 or participant$1)).ti,ab. (71544)
**34**  (assigned or allocated).ti,ab. (98865)
**35**  (controlled adj7 (study or design or trial)).ti,ab. (61542)
**36**  (volunteer or volunteers).ti,ab. (38618)
**37**  human experiment/ (0)
**38**  trial.ti. (41127)
**39**  or/20-38 (1092536)
**40**  (random$ adj sampl$ adj7 ("cross section$" or questionnaire$1 or survey$ or database$1)).ti,ab. not (comparative study/ or controlled study/ or randomi?ed controlled.ti,ab. or randomly assigned.ti,ab.) (3848)
**41**  [cross-sectional study/de not (exp randomized controlled trial/ or controlled clinical trial/ or controlled study/ or randomi?ed controlled.ti,ab. or control group$1.ti,ab.)] (0)
**42**  ((case adj control$).mp. and random$.ti,ab.) not randomi?ed controlled.ti,ab. [mp=title, abstract, heading word, table of contents, key concepts, original title, tests & measures, mesh word] (2092)
**43**  (nonrandom$ not random$).ti,ab. (2035)
**44**  "random field$".ti,ab. (419)
**45**  (random cluster adj3 sampl$).ti,ab. (317)
**46**  (review.ab. and review.pt.) not trial.ti. (0)
**47**  (rat or rats or mouse or mice or swine or porcine or murine or sheep or lambs or pigs or piglets or rabbit or rabbits or cat or cats or dog or dogs or cattle or bovine or monkey or monkeys or trout or marmoset$1).ti. and animal experiment/ (66)
**48**  animal experiment/ not (human experiment/ or human/) (778)
**49**  systematic review.ti,ab. (49888)
**50**  (review.ab. and review.pt.) not trial.ti. (0)
**51**  "we searched".ab. and (review.ti. or review.pt.) (3893)
**52**  "update review".ab. (22)
**53**  (databases adj4 searched).ab. (9036)
**54**  or/40-53 (63268)
**55**  39 not 54 (1064222)
**56**  5 and 8 and 19 and 55 (**113**)
