# Supplementary material for: Effect of Virtual Reality–Based Therapies on Lower Limb Functional Recovery in Stroke Survivors: Systematic Review and Meta-Analysis
Source: J Med Internet Res. 2025 Jul 30;27:e72364. doi: 10.2196/72364 (PMC12310189; doi:10.2196/72364)
Supplement: Multimedia Appendix 2 [file jmir-v27-e72364-s002.pdf]

APPENDIX 2

Baseline Characteristics of Included Studies

Study ID	Study Design	Country	Sample Size	VR Type	VR Devices	Intervention Tool	Intervention Duration	Intervention Frequency	Outcome Measures
Anwar et al., 2021	single blinded randomized clinical trial	Pakistan	68	NIVR	Nintendo Wii	VR training intervention was performed using a Nintendo Wii home video game console. The patient was asked to shift his/her weight while standing on the balance board.The balance board was placed in the middle of the parallel bars to prevent any upper limb assistance that the patient would need if he lost his balance during [illegible]	6 weeks	24	BBS; Fugl-Meyar assessment of the lower extremity (FMA-LE)
Barcala et al., 2013	single blinded randomized clinical trial	Brazil	20	NIVR	Nintendo Wii Balance Board by using the Wii Fit® program	A few minutes of visual biofeedback balance training were performed using the Wii Fit program, which reproduces real movements in virtual actions. The device consists of a platform, known as the Wii Balance Board, which has measurements of weight and center of gravity.	5 weeks	10	BBS; TUG; Independence in activities of daily living; Body symmetry
Bian et al., 2022	single blinded randomized clinical controlled trial	China	44	NIVR	Wii exergame training system, an active and passive trainer with a VR screen, a VR balance training system and a VR gait training system	Wii exergame training system, active and passive trainers with VR screens, VR balance training system, and VR gait training system based on non-immersive VR technology with visual, auditory, and digital feedback.	3 weeks	15	BBS; Stride length; Step size; Fugl-Meyar assessment of the lower extremity (FMA-LE)
Chen et al., 2021	single blinded randomized clinical controlled trial	Taiwan of China	30	NIVR	A screen and a Kinect sensor (Microsoft Corporation, Redmond, WA, USA)	The screen and Kinect sensor are used. The Kinect sensor contains infrared light and a camera to create a three-dimensional (3D) picture of the area in front of it. The device enables full-body 3D motion capture. Participants interacted with the video avatar according to the representation on the TV screen. Game-based tele-rehabilitation programs provide goal-directed exercises in a virtual environment that correspond to the abilities of the [illegible]	4 weeks	12	BBS; TUG; Motricity Index (MI); Functional Ambulation Category (FAC)
de Rooij et al., 2021	single-blinded, randomized controlled trial	Netherlands	52	SIVR	GRAIL consists of an instrumented dual-belt treadmill combined with a motion-capture system (Vicon Motion Systems, Oxford, UK) and a 180° semi-cylindrical screen for the projection of synchronized 3-dimensional environments.	The experimental group was evaluated in the Interactive Laboratory for Real-time Analysis of Gait (GRAIL; Motekforce Link, Amsterdam, the Netherlands). GRAIL consists of an instrumented dual-belt treadmill with a Motion capture system (Vicon Motion Systems, Oxford, UK) and a 180° semi-cylindrical screen for projecting a synchronized 3D environment. To create a safe environment, participants wore seat belts without providing weight support. Various rehabilitation applications (VR environments) with specific rehabilitation goals are available.	6 weeks	12	6-MWT; FES-I; USER-P; TUG (geometric mean, 95%CI); Fatigue severity scale (FSS); Hospital Anxiety and Depression Scale (HADS); Stroke Specific Quality of Life Scale (SS-QOL)
Hung et al., 2014	single-blind randomized controlled trial	Taiwan of China	28	NIVR	The Nintendo Wii Fit	The game balance board, with 4 sensors, is used to evaluate the force distribution and the corresponding movement of the center of pressure (COP). The COP variation was designed as a video game controller. Seven games were selected from the Wii Fit Plus package.	12 weeks	24	TUG; FES-I; Physical Activity Enjoyment Scale (PACES)
In et al., 2016	single-blind randomized controlled trial	Korea	25	SIVR	Virtual reality reflexology (VRRT) equipment. Includes camera and LCD monitor	Movements on the unaffected side were captured with a video camera, enabling the patient to view projected images on a monitor above the affected limb. Using optical illusions to provide a wider field of view and to see both limbs from the same Angle, thus avoiding causing asymmetric postures.	4 weeks	20	BBS; TUG; FRT; PDM multifunctional force measuring plate
Karasu et al., 2018	single-blind randomized-controlled trial	Türkiye	23	NIVR	Wii Fit and Wii Balance Board	Virtual reality exercises for the Nintendo Wii system. The Wii Balance board can sense weight shifts in different directions. By using an avatar to precisely reflect this information on the TV screen, it allows individuals to observe their movements and provide positive feedback. Balance games from the Wii Fit kit were used during the exercise.	4 weeks	20	BBS; TUG; FRT; Postural assessment scale; Walking score
Kim et al., 2009	Double-Blind, Randomized Controlled Study	Korea	24	SIVR	The IREX VR system .The video camera system captures body images, and the subject is then immersed inside a VR scene, interacting with virtual environments and objects.	The IREX VR system was used to enhance gait-related motivation as well as static and dynamic balance performance in stroke patients. This portable VR system includes a television monitor, a video camera, a web glove, and virtual objects, scenes, and large screens. The camera system captures the body image and then immerse the subject in the VR scene to interact with the virtual environment and objects.	4 weeks	16	BBS; 10-MWT; Stride length; Step size; Step frequency; Modified motor assessment scale (MMAS); Computerized balance and gait performance measurements
Krpic et al., 2013	randomized-controlled trial	Slovenia	17	SIVR	Architecture is client/server with two distinct types of clients and a central database.	The experimental group participated in a remote rehabilitation program, which involved balance training using VR tasks and remote diagnosis via a remote computer, tablet, or smartphone.	4 weeks	20	BBS; TUG; 10-MWT
Kržišnik et al., 2021	single-blind randomized-controlled trial	Slovenia	22	SIVR	Zebris Rehawalk® software system (zebris Medical GmbH, Isny im Allgäu, Deutschland) with treadmill.	Participants in EG performed walking training using VR exercises included in the zebris Rehawalk® software system (zebris Medical GmbH, Isny im Allgau, Deutschland). VR feedback training was performed simultaneously with the aid of a large display mounted in front of the treadmill.	4 weeks	20	TUG; 10-MWT; 6-MWT; Functional gait assessment (FGA); Four-square step test (FSST); Step width (SW); The duration of limb support (DS); Cadence and lateral asymmetry (LA) measured during treadmill walking
Kwak et al., 2024	randomized-controlled trial	Korea	36	IVR	The Oculus Quest 2, a head-mounted display (HMD)	FIVR training utilized a head-mounted display (HMD) Oculus Quest 2 for balance training through a commercially available sports game installed on the device. During the training session, participants performed goal-directed upper limb movements while maintaining a standing posture. These movements were designed to elicit postural sway, prompting the desired postural adjustment.	5 weeks	15	BBS; TUG; Stride length; Length of step
Llorens et al., 2015	single-blind randomized-controlled trial	Spain	20	NIVR	A standard computer, an audiovisual output system and a motion tracking system	The hardware setup consisted of a standard computer, an audiovisual output system, and a motion tracking system. The output system consists of a video display and an audio system. The virtual rehabilitation system enables positional audio, thereby providing 3D audio stimuli with appropriate speaker configuration.	4 weeks	20	BBS; 10-MWT; Step Brunel balance assessment; Tinetti Performance
McEwen et al., 2014	Double-Blind, Randomized Controlled trial	Canada	59	NIVR	Interactive Rehabilitation Exercise software (IREX; GestureTek; Toronto, Ontario, Canada; for a detailed description of individual games, see Methods in the online-only Data Supplement)	Participants in the experimental group interacted in a standing position with VR games (e.g., soccer goalkeeping, snowboarding) that challenged their balance and weight transfer.	3 weeks	21	TUG; 2-MWT; Stroke Assessment Scale Leg Domain (CMSA-Leg)
Mirelman et al., 2009	single-blind randomized-controlled trial	America	18	NIVR	Rutgers Ankle Rehabilitation System, a 6-degree of freedom Stewart platform force-feedback system that allows individuals to exercise the lower extremity by navigating through a virtual environment that is displayed on a desktop computer.	Subjects in the robotic VR group performed exercises by using foot movements to navigate an aircraft or boat in a virtual environment containing a series of targets. Target position and timing were manipulated to ensure that training included discrete and combined ankle movements.	4 weeks	12	Step size; Walking distance; Speed of step

Park et al., 2013	single-blind randomized-controlled trial	Korea	16	IVR	Not listed	The VR based postural control program consists of a program to improve gait ability by visual feedback compared to the reference motion scene and real motion. The program consisted of three phases: trunk stabilization and pelvic tilt in the supine position.	4 weeks	12	10-MWT; Stride length; Step size; Functional Walking curve
Peláez-Vélez et al., 2023	single-blind randomized-controlled trial	Spain	24	IVR	An immersive VR by means of VR glasses (Oculus Quest 2), a computer (with the software), a camera (Kinect 360 v1) used as a sensor of the patient's movements and a router that acted as a connection point between the glasses and the	The VR program applied was via VR goggles (Oculus Quest 2), a computer (with software), a camera used as a patient motion sensor (Kinect 360 v1), and a router used as a connection point between the goggles and the computer.	6 weeks	30	BBS; Modified Ashworth scale (MAS); Trunk control test; Tinetti balance scale; Functional walking classification of the Sagunto Hospital
Sana et al., 2023	single-blind randomized-controlled trial	Pakistan	30	NIVR	Nintendo Wii console (RVL001, Nintendo, EUR), Wii Balance Board, Nintendo Wii remote control (RVL-003), and Wii Fit Plus software (Nintendo D-63760 GroBostheim)	The VR training program includes the Nintendo Wii console (RVL001, Nintendo, Euro), Wii Balance Board, Nintendo Wii Remote (RVL-003), and Wii Fit Plus software (Nintendo D-63760 GroBostheim).	8 weeks	24	TUG; DGI; Dizziness Disorder Inventory (DHI)
Singh et al., 2013	single-blind randomized-controlled trial	Malaysia	28	NIVR	Nintendo® Wii Fit Plus and Xbox 360 Kinect	The VR games performed were Balance Bubble using Nintendo® Wii Fit Plus and Balance Board and Rally Ball using Xbox 360 Kinect for 15 minutes each. A therapeutic assistant supervised the participants in a one-on-one manner during the VR game.	6 weeks	12	TUG; 10-MWT; Barthel index
Song & Park, 2015	randomized-controlled trial	Korea	40	NIVR	Xbox Kinect	The experimental group used Xbox Kinect to perform each training session for 30 minutes, five sessions several times a week for 8 weeks. In front of the Xbox Kinect sensor, subjects moved their bodies to play the game. Various games such as Kinect Sport, Kinect Sport Season 2, Kinect Adventure, and Kinect Gunstringer were designed to stimulate the subjects' interest and prevent boredom.	8 weeks	40	TUG; 10-MWT; Limit of stability (LOS); Beck Depression Inventory (BDI); Relationship Change Scale (RCS)
Sultan et al., 2023	double-blind randomized control trial	Pakistan	40	NIVR	Xbox Kinect	Xbox Kinect sports games	8 weeks	24	BBS; TUG; Functional independence measure (FIM); Trunk Injury Scale (TIS)
Wang et al., 2024	randomized-controlled trial	China	54	SIVR	C-Mill (ForceLink, Culemborg, the Netherlands) is an intelligent treadmill that combines VR with augmented reality technology.	The C-Mill (ForceLink, Kullenborg, the Netherlands) is a smart treadmill that combines VR with augmented reality technology. Vr can be used to immert patients in a virtual environment and improve their walking compliance, or a projector can be used to alter the visual environment by projecting obstacles onto the treadmill to trigger gait adjustments by the user.	5 weeks	25	TUG; Walking speed; obstacle avoidance ability; center of pressure (COP); Barthel Index (BI)
Yatar & Yildirim, 2015	randomized-controlled trial	Cyprus	30	NIVR	Nintendo Wii Fit	To compare the effectiveness of gaming technology based Wii Fit balance training (WBT) with progressive balance training (PBT) and the Neurodevelopmental Therapy Program static and dynamic balance functions, activity specific balance confidence, and activity in daily life of chronic stroke patients.	4 weeks	12	BBS; TUG; FRT; DGI
Yom et al., 2015	single-blind randomized-controlled trial	Korea	20	SIVR	Notebook computer (X-note 280; LG, Korea), beam projector (PLC-XW55; Sanyo, Japan), and screens.	The experimental group adopted the virtual reality based ankle exercise (VRAE), which consisted of four exercise items: exercise on the floor, exercise on the balance board, exercise on the padded ball, and standing on one foot. All subjects remained in a standing position during the exercise. The virtual reality environment was created using a virtual reality-based ankle exercise program, a laptop computer and screen.	6 weeks	30	TUG; Stride length; Step size; Gait speed; Modified Ashworth Scale (MAS)

Abbreviations: IVR: Immersive VR; SIVR: Semi-immersive VR; NIVR: Non-immersive VR; BBS: Berg Balance Scale; TUG: Timed Up and Go Test; 10-MWT: 10-Meter Walk Test; FRT: Functional Reach Test; FES-I: Falls Efficacy Scale-International; DGI: Dynamic Gait Index

Participants Demographic of Included Studies

Study ID	Male (proportion)	Mean Age in EG	Mean Age in CG	Time after Stroke (months)	Lesion Side(right/left)	Stroke type (infarction/hemorrhage) in EG	Stroke type (infarction/hemorrhage) in CG
Anwar et al., 2021	50.0%	51.56±7.19	51.35±5.78	Not mentioned	Not mentioned	Not mentioned	Not mentioned
Barcala et al., 2013	45.0%	65.2±12.5	63.5±14.5	EG: 12.3±7.1<br>CG: 15.2±6.6	13/7	9/1	9/1
Bian et al., 2022	79.4%[$]	53.25±8.72[$]	55.00±10.27[$]	<6	Not mentioned	Not mentioned	Not mentioned
de Rooij et al., 2021	69.2%	65 (57–70)	61 (53–71)	EG: 84 (69–110)<br>CG: 66 (51–103)<br>(day)	Not mentioned	24/20	4/4
Hung et al., 2014	64.3%	55.38±9.95	53.40±10.03	EG: 21.00±11.26<br>CG: 15.93±8.02	19/9	7/6	9/6
Chen et al., 2021	60.0%	61 (53–68)	60 (52–68)	EG: 2.5 (1.08–5.17)<br>CG: 1.5 (1.08–2.33)<br>(year)	14/16	8/7	9/6
Wang et al., 2024	72.2%	62 (16)	58 (19)	EG: 4 (7.00)<br>CG: 2 (2.00)	27/27	20/7	22/5
In et al., 2016	60.0%	57.31±10.53	54.42±11.44	EG: 12.54±4.14<br>CG: 13.58±5.28	12/13	8/5	8/4
Karasu et al., 2018	43.5%	62.3 (11.79)	64.1 (12.2)	EG: 29 (14–348)<br>CG: 31 (13–324)<br>(day)	Not mentioned	8/4	10/1
Kim et al., 2009	58.3%	52.42±10.09	51.75±7.09	EG: 25.91±9.96<br>CG: 24.25±8.87	13/11	Not mentioned	Not mentioned
Krpic et al., 2013	64.7%	58.5±12.1	63.0±8.5	EG: 3-8<br>CG: 2-10	8/9	Not mentioned	Not mentioned
Kržišnik et al., 2021	68.2%	59.8±7.7	54.7±6.0	EG: 5.0±2.4<br>CG: 4.5±2.0	15/7	10/1	10/1
Kwak et al., 2024	58.3%	54.28±17.74	59.17±13.86	EG: 26.00±12.04<br>CG: 27.61±10.27	19/17	2/16	4/14
Llorens et al., 2015	45.0%	58.3 ±11.6	55.0 ±11.6	>6	Not mentioned	7/3	6/4
McEwen et al., 2014	54.2%	62.2±14.1	66.0±15.8	Not mentioned	31/21<br>(Bilateral: 7)	23/7	25/4

Mirelman et al., 2009	83.3%	61.8±9.94	61±8.32	EG: 37.7±25.0<br>CG: 58.2±26.3	8/10	Not mentioned	Not mentioned
Park et al., 2013	68.8%	46.25±6.84	48.75±8.81	EG: 11.63±4.44<br>CG: 11.25±4.53<br>(year)	8/8	5/3	4/4
Peláez-Vélez et al., 2023	66.7%	51.91±18.58	59.58±15.97	>6	13/11	Not mentioned	Not mentioned
Sana et al., 2023	43.3%	49.73±5.81	54.87±9.47	1-6	14/16	Not mentioned	Not mentioned
Singh et al., 2013	57.1%	65.4±9.8	67.0±8.4	EG: 40.5 ± 41.8<br>CG: 34.9 ± 23.6	15/13	Not mentioned	Not mentioned
Song & Park, 2015	55.0%	51.37±40.6	50.10±7.83	EG: 14.75±6.06<br>CG: 14.30±3.40	19/21	Not mentioned	Not mentioned
Sultan et al., 2023	77.5%	58.6±3.3	58.1±4.3	EG: 7.2±1.8<br>CG: 6.3±2.1	27/13	18/2	18/2
Yatar & Yildirim, 2015	43.3%	62.80±10.87	56.6±16.42	EG: 3.70±4.42<br>CG: 4.23±4.86<br>(year)	15/15	Not mentioned	Not mentioned
Yom et al., 2015	55.0%	64.60	78.10	EG: 11.14<br>CG: 11.63	14/6	Not mentioned	Not mentioned

$^{\$}$demographic characteristics at one-year follow-up; data on demographic characteristics after the intervention were not available
EG: Experimental group; CG: Control group
Values expressed as Mean±SD or Median(IQR)
