# Supplementary material for: Effect of Virtual Reality–Based Therapies on Lower Limb Functional Recovery in Stroke Survivors: Systematic Review and Meta-Analysis
Source: J Med Internet Res. 2025 Jul 30;27:e72364. doi: 10.2196/72364 (PMC12310189; doi:10.2196/72364)

APPENDIX 3

Figure 1. Sensitivity analysis of virtual reality effects on balance (measured by Berg Balance Scale), based on different virtual reality devices. VR: virtual reality; BBS: Berg Balance Scale.

	VR therapy			Conventional therapy				Mean Difference
**Study or Subgroup**	**Mean**	**SD**	**Total**	**Mean**	**SD**	**Total**	**Weight**	**IV, Random, 95% CI**
**1.2.1 Nintendo Wii**								
Anwar et al., 2021	25.71	8.13	34	11.29	6.65	34	9.6%	14.42 [10.89, 17.95]
Barcala et al., 2013	2.3	6.68	10	5	5.02	10	8.1%	−2.70 [−7.88, 2.48]
Bian et al., 2022	1.44	9.76	23	−2.42	13.62	21	6.5%	3.86 [−3.20, 10.92]
Karasu et al., 2018	10.1	6.66	12	3.1	6.66	11	7.9%	7.00 [1.55, 12.45]
Yatar & Yildirim, 2015	4.73	4.78	15	5.2	8.54	15	8.4%	−0.47 [−5.42, 4.48]
**Subtotal (95% CI)**			**94**			**91**	**40.6%**	**4.54 [−2.54, 11.62]**
Heterogeneity: $Tau^2 = 57.94$; $Chi^2 = 39.62$, df = 4 (P < 0.00001); $I^2 = 90\%$								
Test for overall effect: Z = 1.26 (P = 0.21)								
**1.2.2 Xbox Kinect**								
Peláez-Vélez et al., 2023	19	14.69	12	7.58	21.68	12	2.7%	11.42 [−3.40, 26.24]
Sultan et al., 2023	6.9	4.8	20	4	6.61	20	9.6%	2.90 [−0.68, 6.48]
**Subtotal (95% CI)**			**32**			**32**	**12.2%**	**4.00 [−1.60, 9.61]**
Heterogeneity: $Tau^2 = 6.05$; $Chi^2 = 1.20$, df = 1 (P = 0.27); $I^2 = 17\%$								
Test for overall effect: Z = 1.40 (P = 0.16)								
**1.2.3 Others**								
Chen et al., 2021	5	13.39	15	4	10.91	15	5.3%	1.00 [−7.74, 9.74]
In et al., 2016	3.62	3.63	13	1.33	3	12	10.3%	2.29 [−0.31, 4.89]
Kim et al., 2009	6.75	5.29	12	1.58	4.01	12	9.4%	5.17 [1.41, 8.93]
Krpic et al., 2013	5.5	14	6	12.55	11.93	11	3.2%	−7.05 [−20.29, 6.19]
Kwak et al., 2024	1.38	2.53	18	0.83	2.16	18	11.0%	0.55 [−0.99, 2.09]
Llorens et al., 2015	3.8	5.94	10	1.8	6.45	10	7.9%	2.00 [−3.43, 7.43]
**Subtotal (95% CI)**			**74**			**78**	**47.2%**	**1.79 [−0.00, 3.59]**
Heterogeneity: $Tau^2 = 1.39$; $Chi^2 = 7.12$, df = 5 (P = 0.21); $I^2 = 30\%$								
Test for overall effect: Z = 1.96 (P = 0.05)								
**Total (95% CI)**			**200**			**201**	**100.0%**	**3.29 [0.52, 6.06]**
Heterogeneity: $Tau^2 = 17.58$; $Chi^2 = 63.47$, df = 12 (P < 0.00001); $I^2 = 81\%$								
Test for overall effect: Z = 2.32 (P = 0.02)								
Test for subgroup differences: $Chi^2 = 1.01$, df = 2 (P = 0.60), $I^2 = 0\%$								

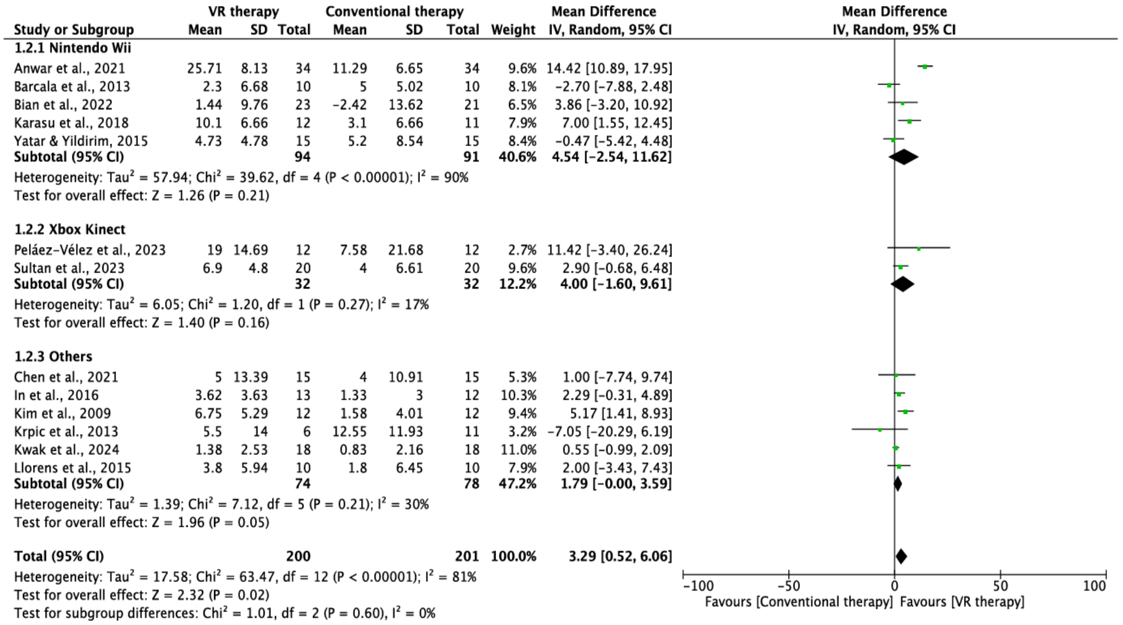

Supplement: Multimedia Appendix 3 [file jmir-v27-e72364-s003.pdf]
