# Supplementary material for: Effect of Virtual Reality–Based Therapies on Lower Limb Functional Recovery in Stroke Survivors: Systematic Review and Meta-Analysis
Source: J Med Internet Res. 2025 Jul 30;27:e72364. doi: 10.2196/72364 (PMC12310189; doi:10.2196/72364)

APPENDIX 4

Figure S1. Subgroup analysis of VR effects on gait speed by time after stroke (measured by 10-MWT). VR: virtual reality; 10-MWT: 10-meter walk test; CI: confidence interval.

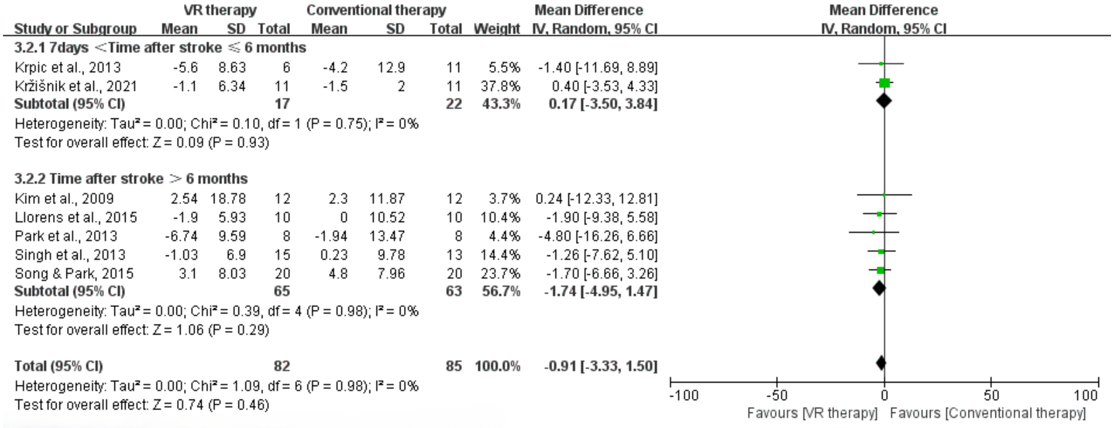

Figure S2. Subgroup analysis of VR effects on gait speed by VR type (measured by 10-MWT). VR: virtual reality; 10-MWT: 10-meter walk test; CI: confidence interval.

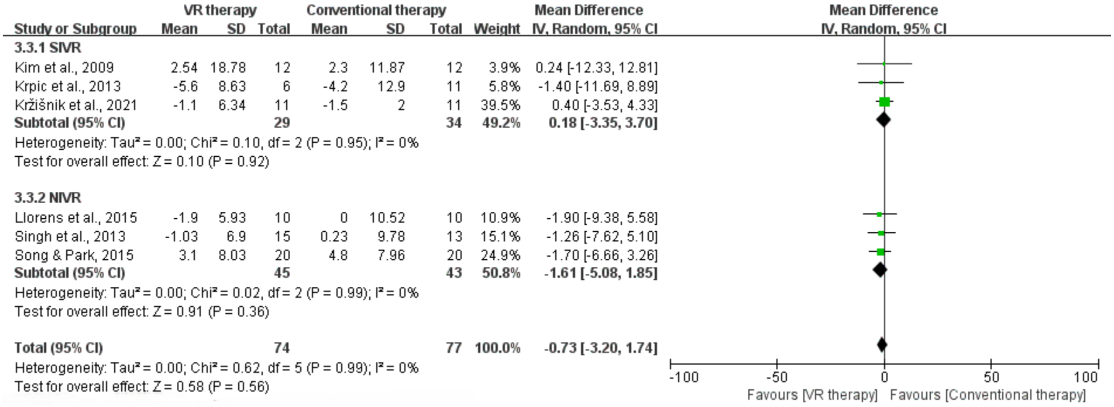

Figure S3. Subgroup analysis of VR effects on gait speed by total frequency (measured by 10-MWT). VR: virtual reality; 10-MWT: 10-meter walk test; CI: confidence interval.

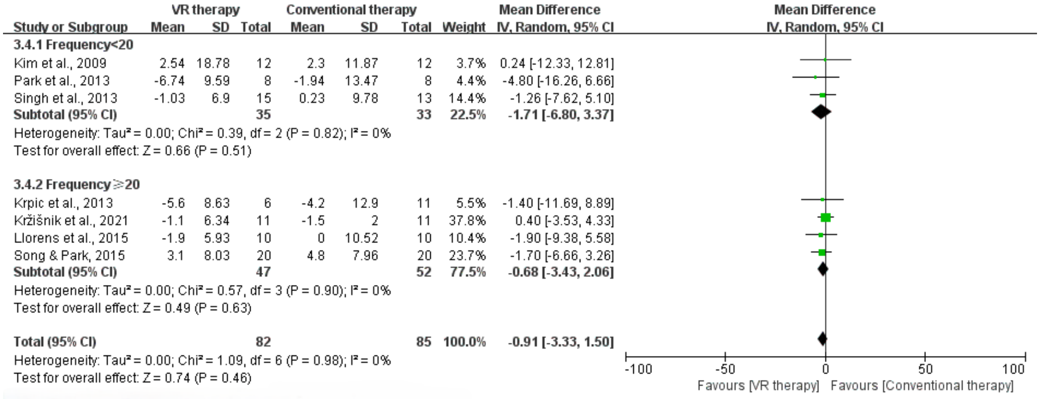

Figure S4. Effect of VR therapy on stride length. VR: virtual reality; CI: confidence interval.

Study or Subgroup	VR therapy Mean	VR therapy SD	VR therapy Total	Conventional therapy Mean	Conventional therapy SD	Conventional therapy Total	Weight	Mean Difference IV, Random, 95% CI
Bian et al., 2022	11	20.52	23	6	21.07	21	26.7%	5.00 [-7.31, 17.31]
Kim et al., 2009	6.66	16.33	12	1.26	11.08	12	32.4%	5.40 [-5.77, 16.57]
Kwak et al., 2024	8	25.18	18	3.11	20.32	18	18.1%	4.89 [-10.06, 19.84]
Park et al., 2013	11.78	19.4	8	3.45	23.14	8	9.2%	8.33 [-12.59, 29.25]
Yom et al., 2015	9.32	23.03	10	2.76	15.63	10	13.6%	6.56 [-10.69, 23.81]
**Total (95% CI)**			**71**			**69**	**100.0%**	**5.63 [-0.73, 11.99]**

Heterogeneity: Tau² = 0.00; Chi² = 0.10, df = 4 (P = 1.00); I² = 0%
Test for overall effect: Z = 1.74 (P = 0.08)

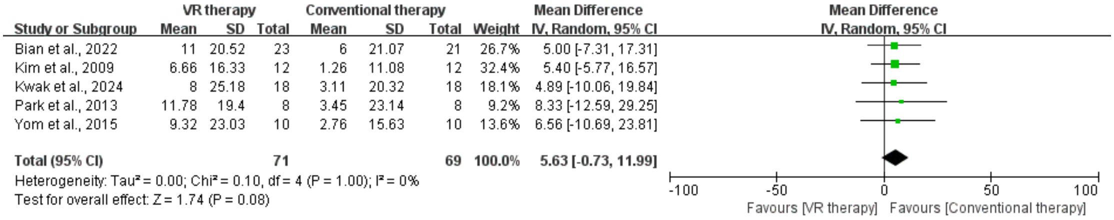

Figure S5. Effect of VR therapy on dynamic balance (measured by FRT). VR: virtual reality; FRT: functional reach test; CI: confidence interval.

Study or Subgroup	VR therapy Mean	VR therapy SD	VR therapy Total	Conventional therapy Mean	Conventional therapy SD	Conventional therapy Total	Weight	Mean Difference IV, Random, 95% CI
In et al., 2016	0.67	5.89	13	-0.1	7.1	12	26.9%	0.77 [-4.37, 5.91]
Karasu et al., 2018	8.8	5.5	12	3.4	4.48	11	38.0%	5.40 [1.31, 9.49]
Yatar & Yildirim, 2015	4.14	5.58	15	2.93	6.44	15	35.1%	1.21 [-3.10, 5.52]
**Total (95% CI)**			**40**			**38**	**100.0%**	**2.68 [-0.30, 5.67]**

Heterogeneity: Tau² = 1.77; Chi² = 2.68, df = 2 (P = 0.26); I² = 25%
Test for overall effect: Z = 1.76 (P = 0.08)

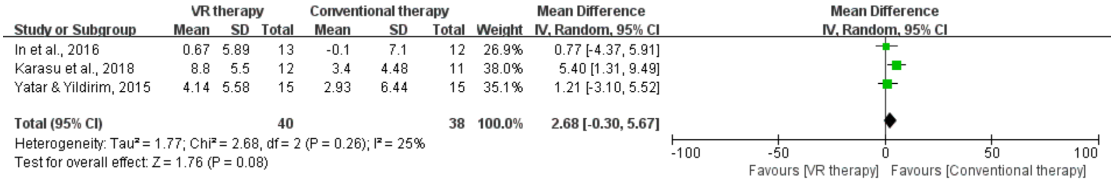

Figure S6. Effect of VR therapy on dynamic balance (measured by DGI). VR: virtual reality; DGI: dynamic gait index; CI: confidence interval.

Study or Subgroup	VR therapy Mean	VR therapy SD	VR therapy Total	Conventional therapy Mean	Conventional therapy SD	Conventional therapy Total	Weight	Mean Difference IV, Random, 95% CI
Sana et al., 2023	5.9	2.2	15	4.4	2.78	15	69.3%	1.50 [-0.29, 3.29]
Yatar & Yildirim, 2015	2	3.79	15	1.86	3.74	15	30.7%	0.14 [-2.55, 2.83]
**Total (95% CI)**			**30**			**30**	**100.0%**	**1.08 [-0.41, 2.58]**

Heterogeneity: Tau² = 0.00; Chi² = 0.68, df = 1 (P = 0.41); I² = 0%
Test for overall effect: Z = 1.42 (P = 0.16)

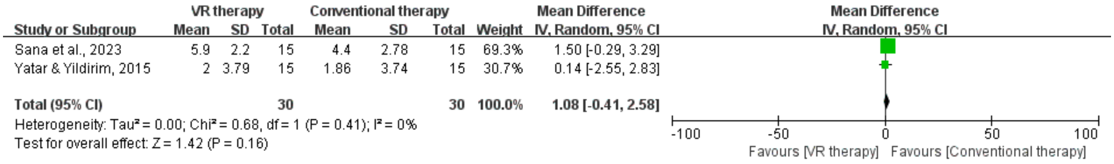

Figure S7. Effect of VR therapy on fear of falling (measured by FES-I). VR: virtual reality; FES-I: falls efficacy scale-international; CI: confidence interval.

Study or Subgroup	VR therapy Mean	VR therapy SD	VR therapy Total	Conventional therapy Mean	Conventional therapy SD	Conventional therapy Total	Weight	Mean Difference IV, Random, 95% CI
de Rooij et al., 2021	20.96	9.57	28	20.75	5.12	24	56.5%	0.21 [-3.88, 4.30]
Hung et al., 2014	-3.77	6.49	13	-3.87	6.04	15	43.5%	0.10 [-4.57, 4.77]
**Total (95% CI)**			**41**			**39**	**100.0%**	**0.16 [-2.92, 3.24]**

Heterogeneity: Tau² = 0.00; Chi² = 0.00, df = 1 (P = 0.97); I² = 0%
Test for overall effect: Z = 0.10 (P = 0.92)

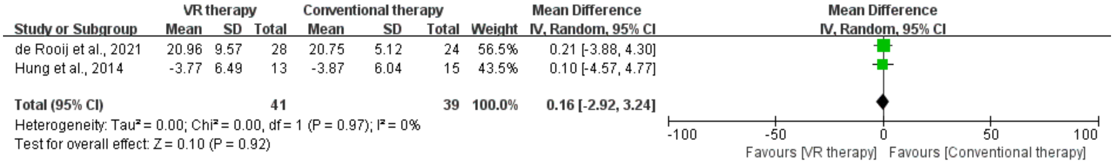

Supplement: Multimedia Appendix 4 [file jmir-v27-e72364-s004.pdf]
