# Supplementary figures and images for: Effect of Virtual Reality–Based Therapies on Lower Limb Functional Recovery in Stroke Survivors: Systematic Review and Meta-Analysis
Source: J Med Internet Res. 2025 Jul 30;27:e72364. doi: 10.2196/72364 (PMC12310189; doi:10.2196/72364)

## APPENDIX 5

Funnel plot of publication bias.

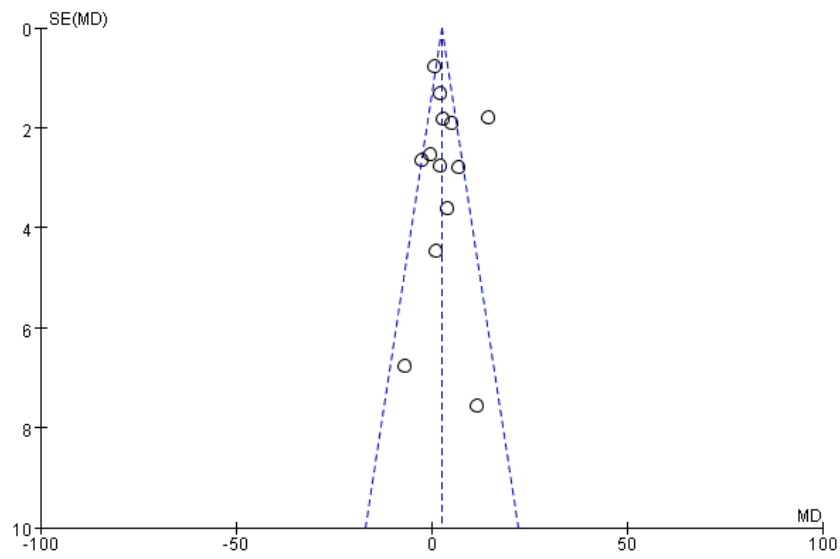

Supplement: Multimedia Appendix 5 [file jmir-v27-e72364-s005.pdf]
